# Spatial Evolution of Coke in ZSM‐5 Catalysts During Methanol‐to‐Hydrocarbons Conversion Revealed by In Situ X‐Ray Photoelectron Spectroscopy

**DOI:** 10.1002/anie.7876229

**Published:** 2026-06-13

**Authors:** Luca Artiglia, Hannes Frey, Loïc Bénariac‐Doumal, Sung Sik Lee, Przemyslaw Rzepka, Jeroen A. van Bokhoven, Vladimir Paunović

**Affiliations:** ^1^ PSI Center for Energy and Environmental Sciences Paul Scherrer Institute Villigen Switzerland; ^2^ Institute For Chemical and Bioengineering Department of Chemistry and Applied Biosciences ETH Zurich Zurich Switzerland; ^3^ J. Heyrovsky Institute of Physical Chemistry Czech Academy of Sciences Prague Czech Republic; ^4^ Scientific Center for Optical and Electron Microscopy (ScopeM) Zurich Switzerland

**Keywords:** catalyst coking, in situ x‐ray photoemission spectroscopy, methanol‐to‐hydrocarbons conversion, reaction mechanism, zeolites

## Abstract

Along with assessing chemical pathways, understanding the spatial evolution of deactivating coke species in zeolites is essential for enhancing their catalytic efficiency in processes for chemical and fuel production. In this study, synchrotron‐based in situ X‐ray photoemission spectroscopy experiments provide insights into the coke deposition on ZSM‐5 catalysts with different Si/Al ratios during methanol‐to‐hydrocarbons conversion, which is an industrially relevant reaction. Surface‐sensitive C 1s X‐ray photoemission spectroscopy with a probing depth ≈1 nm demonstrates that the less acidic ZSM‐5 zeolites exhibit a more pronounced coke accumulation on crystal surfaces and promote the formation of highly condensed, graphite‐like structures than the higher acidic ones. Analysis of coking kinetics, lattice deformation, and coke composition using Raman, in situ infrared, and *operando* UV‐vis spectroscopies further supports enhanced surface coking in low‐acidity, slowly deactivating catalysts and more prominent micropore coke formation in high‐acidity, fast‐deactivating catalysts. The results indicate that acid site density governs both the kinetics and the preferential location of coke, shedding light on the fundamental nanoscale processes underlying coke‐induced deactivation of zeolite catalysts.

## Graphical abstract

Coking is the central source of deactivation of zeolite catalysts. Using in situ XPS along with complementary methods, we identify differences in the formation kinetics and composition of surface coke on industrially relevant ZSM‐5 catalysts during MTH conversion, demonstrating the strong potential of this technique for studying catalyst deactivation due to coking.

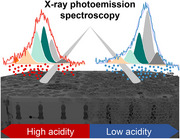

## 1 Introduction

Zeolites are indispensable industrial catalysts in oil refining and processes for the valorization of biomass, plastics, and C_1_ feedstocks [1, 2, 3, 4]. Their catalytic activity arises primarily from the activation of organic molecules over Brønsted (BAS) and Lewis (LAS) acid sites, enhanced by the spatial confinement within their micropores. However, the performance of zeolite catalysts is generally constrained by deactivation [1, 2, 3, 4, 5, 6, 7, 8, 9]. Coking, involving the formation of high molecular weight byproducts that block the active sites and micropore volume, is a prevalent deactivation mechanism that significantly impacts industrial processes [1, 6, 9, 10, 11]. A well‐known technology affected by coke formation is the methanol‐to‐hydrocarbons (MTH) conversion [12, 13, 14]. MTH processes enable the transformation of methanol derived from coal, natural gas, or renewable sources into light alkenes, aromatics, and gasoline‐range products, offering a route to sustainable chemicals and fuels. The MTH conversion proceeds via a complex mechanism, in which ethene and higher hydrocarbons are primarily formed through sequential methylation and cracking of the micropore‐confined alkene‐ and arene‐based hydrocarbon pool (HP) carriers over BAS. These reaction sequences, however, extend to the formation of HP‐inactive coke species, mostly composed of polycyclic aromatic hydrocarbons (PAHs), leading to progressive activity loss [15, 16, 17, 18, 19, 20, 21, 22, 23, 24, 25, 26]. Consequently, MTH processes involve catalyst regeneration via coke oxidation [9, 13, 14]. Regeneration converts coke into CO_2_, potentially accounting for up to 8% of the total feedstock [27], and promotes catalyst degradation due to steam evolution at high temperatures [28].

The formation of PAHs is associated with the overgrowth of the arene HP species and condensation of unsaturated molecules, coupled with cyclization and hydrogen‐transfer [8, 15, 16, 21, 23]. These reactions, which are promoted by specific intermediates, such as formaldehyde and polyenes, are primarily catalyzed by BAS and controlled by diffusion and confinement of coke precursors [17, 18, 20, 23, 24, 25, 26]. Specifically, higher BAS density and longer diffusion lengths impose a longer effective residence time of coke precursors [29], thereby increasing the probability of coke formation in the micropores [10]. This so‐called internal coke deposits in regions that provide minimal constraint to its growth modify the micropore geometry and thus the unit cell dimensions [30, 31, 32, 33]. However, the outer crystallite surface contains acid sites and provides a virtually constraint‐free environment for the growth of thermodynamically favored, graphene‐like PAHs [10, 17, 34, 35, 36]. This so‐called external coke obstructs the active volume, even when internal coking is greatly suppressed. This raises the question to which extent external and internal coke deposits contribute to zeolite deactivation and how this depends on zeolite properties, particularly acidity. The distinction between external and internal coke has primarily been based on differences in conjugation length and molecular weight, identified through spectroscopic and mass‐spectrometric methods [10, 17, 34, 35]. However, high‐molecular‐weight PAHs can also develop within the micropores [8]. Relative changes in internal coke content are reflected in changes of lattice parameters observed by X‑ray diffraction, while electron and neutron diffraction enable mapping of coke location and average structure [30, 31, 32, 33]. X‐ray photoemission spectroscopy (XPS) provides highly surface sensitive information on the composition and oxidation state of elements, particularly when combined with synchrotron x‐ray sources (down to ≈0.5–1 nm). In situ XPS measurements in the mbar range offer exceptional possibilities for resolving the composition and structure of reactive catalytic surfaces [37, 38]. However, this technique has not yet been applied to investigate coke formation over zeolites.

In this study, we unveil the differences in external coking over prototypical ZSM‐5 catalysts in the MTH reaction as a function of their acidity by in situ XPS and complemented by diffraction and spectroscopic studies.

## 2 Results and Discussion

ZSM‐5 zeolites Z_15_ and Z_40_, with Si/Al ratios of 15 and 40, respectively, were selected to study coke accumulation due to their industrial relevance and common use as benchmark MTH catalysts [10, 12, 14, 17, 18, 19, 22, 24, 26, 28, 34, 35, 39]. The particle size, crystalline, textural, and diffusion properties of these materials are similar and consistent with those reported in previous studies (Table S1, Figure S1) [26, 28]. ^27^Al MAS NMR spectra show that tetrahedral framework aluminum predominates in both zeolites. ^29^Si MAS NMR and XPS analysis show that framework and surface Si/Al ratios are comparable to the bulk values. Pyridine adsorption confirms a higher concentration of BAS and LAS in Z_15_ than in Z_40,_ while adsorption of 2,6‐di‐*tert*‐butylpyridine indicates that differences in BAS concentration are also preserved in the surface region of the catalyst (Figure S1, Table S1).

**FIGURE 1:**
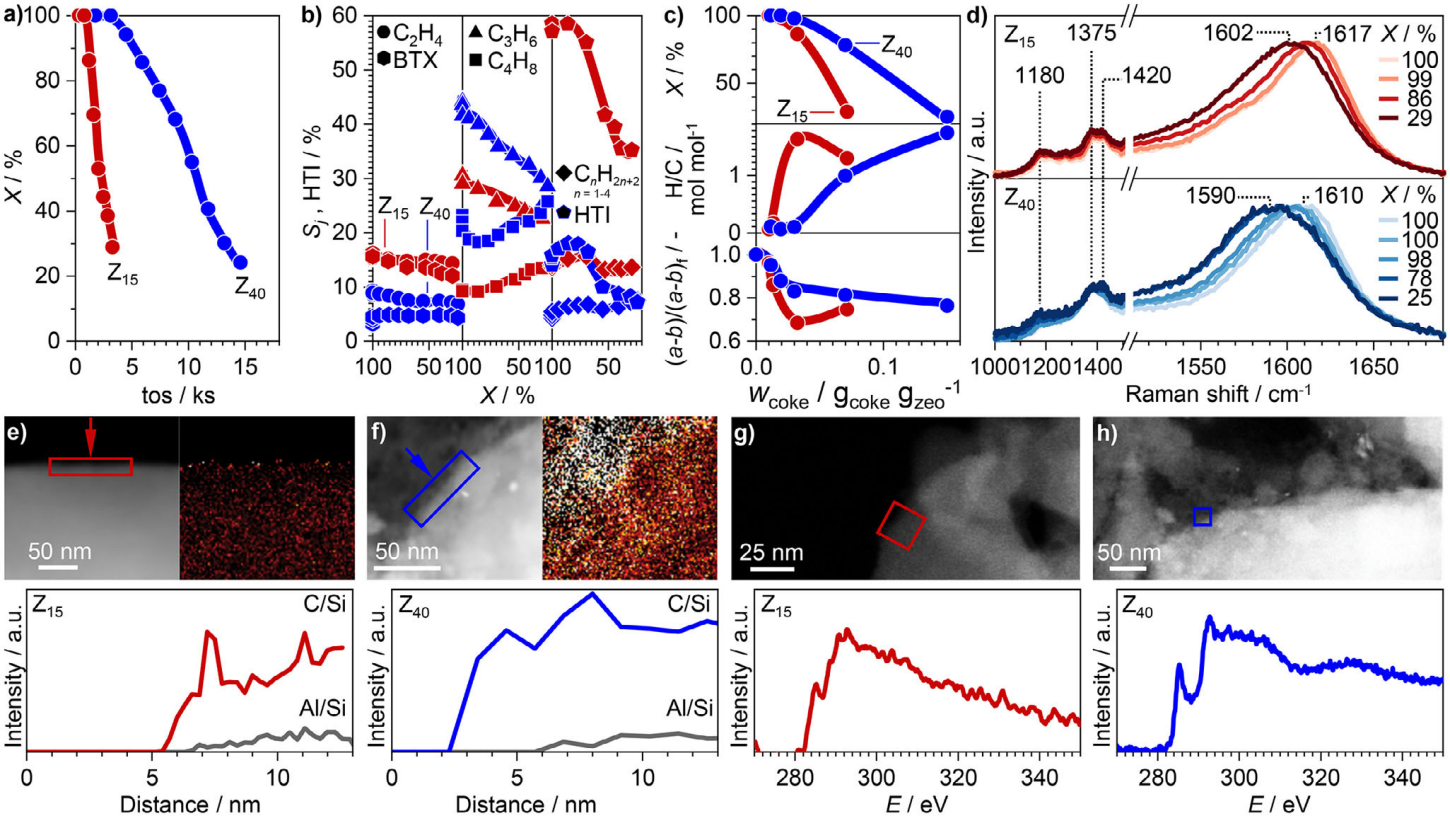
(a) Conversion (*X*) versus time‐on‐stream (tos), and (b) selectivity to selected products (*S_j_*) and hydrogen‐transfer index (HTI) versus conversion in the MTH reaction over Z_15_ and Z_40_ zeolites. (c) Conversion, molar hydrogen‐to‐carbon (H/C) ratio, relative lattice expansion ((*a* − *b*):(*a* − *b*)_fresh_), and weight fraction of coke (*w*_coke_), and (d) Raman spectra of Z_15_ and Z_40_ at different deactivation levels. HAADF‐STEM micrographs and EDXS analysis of deactivated (e) Z_15_ (*X =* 29%) and (f) Z_40_ (*X =* 25%). The EDXS data are shown as C signal heatmaps normalized to Si, and as C/Si and Al/Si profiles along the rectangles in the micrographs following the arrow directions. HAADF‐STEM and normalized EELS spectra of the C K‐edge acquired at the vacuum‐surface interface of deactivated (g) Z_15_ (*X =* 29%) and (h) Z_40_ (*X = *25%), indicated by the squares in the micrographs. Reaction conditions: CH_3_OH:Ar = 19:81 mol%, WHSV = 76 g_CH3OH_ g_cat_^−1^ h^−1^, *T* = 773 K, and *P* = 1.8 bar.

As previously reported [22, 26, 28, 39], Z_15_ deactivates faster than Z_40_, providing *≈* 5.5 × lower cumulative turnover values at 773 K (CT_0_ *≈* 45 vs. CT_0_ *≈* 240 g_CH3OH_^−1^ g_cat_^−1^, Figure 1a). Z_15_ also yields higher selectivity to ethene and benzene, toluene, and xylene (BTX), and lower selectivity to propene and butenes (Figure 1b), which indicates that arene‐mediated reactions are more prevalent in Z_15_ than in Z_40_ [12, 14]. Additionally, Z_15_ exhibits a higher hydrogen‐transfer index (HTI), pointing to enhanced hydrogen‐transfer reactions [18, 19, 26]. The differences in stability and product distribution align with the proposed coking mechanism, indicating that active HP intermediates convert into inactive PAH coke species mainly through overgrowth of arene‐chain carriers coupled with HT reactions [10, 17, 18, 19, 26]. Given the comparable crystal sizes and diffusion properties of Z_15_ and Z_40_, higher BAS density of the former catalyst leads to a longer BAS‐based intracrystalline contact time of the coking precursors [20, 29], which is expected to enhance coke formation in the micropores. In contrast, the shorter BAS‐based residence time in Z_40_ enhances the probability that these species exit micropores. However, they can still react on the outer catalyst surface, forming external coke. Notably, for a similar level of residual conversion, Z_15_ accommodates less coke on a weight basis than Z_40_ (Figure 1c). While the H/C molar ratio of coke increases with its accumulation on both catalysts, reflecting the progressive occlusion of lighter hydrocarbons, it remains lower for Z_40_ at similar coke levels (Figure 1c), indicating a higher degree of coke condensation in this less acidic material [10]. For similar coke content, Z_15_ displays higher deformation of a unit cell, measured as the relative change of a difference between *a* and *b* unit cell parameters with respect to the fresh catalyst (Figure 1c, Figure S2, Table S2) [30]. Assuming the deformation primarily originates from molecules within micropores, this observation supports a greater accumulation of internal coke in more acidic Z_15_ compared to Z_40_. Raman spectra of deactivated catalysts show qualitatively similar features associated with PAHs, with prominent bands at 1185 and 1375 cm^−1^ ascribed to methylated PAHs and lower molecular weight aromatics, respectively, as well as the bands 1420 cm^−1^ and 1590–1620 cm^−1^ also characteristic of ring‐stretches in PAHs (Figure 1d) [21, 40]. The most intense band at 1590–1620 cm^−1^ is consistently shifted to lower frequencies in deactivated Z_40_ as compared to the Z_15_ catalyst, which suggests a higher prevalence of graphene‐like species in the less acidic catalyst. The STEM‐EDX heatmap of the silicon‐normalized carbon signal of highly deactivated Z_40_ shows a much higher intensity at the outer periphery compared to Z_15_, which suggests a difference not fully accounted for by the bulk carbon content and indicates the presence of a thicker layer of surface coke in Z_40_ (Figure 1e,f). Moreover, the carbon K edge EELS acquired at the perimeter of highly deactivated Z_40_ displays more intense and better resolved peaks at ≈286 and 292 eV, corresponding to electron excitations from 1s to antibonding π* and σ* orbitals, respectively [41], than the highly deactivated Z_15_ (Figure 1g,h). This suggests a more ordered, graphitic nature of coke in Z_40_ compared to the more amorphous structure of coke in Z_15_. Consistently, in situ FTIR analysis suggests faster formation of aromatics in Z_15_, and the development of more condensed aromatics in Z_40_, while *operando* DR‐UV‐vis spectroscopy indicates a more prevalent growth of higher PAHs species associated with external coke in Z_40_ than in Z_15_ (Figure S3).

**SCHEME 1:**
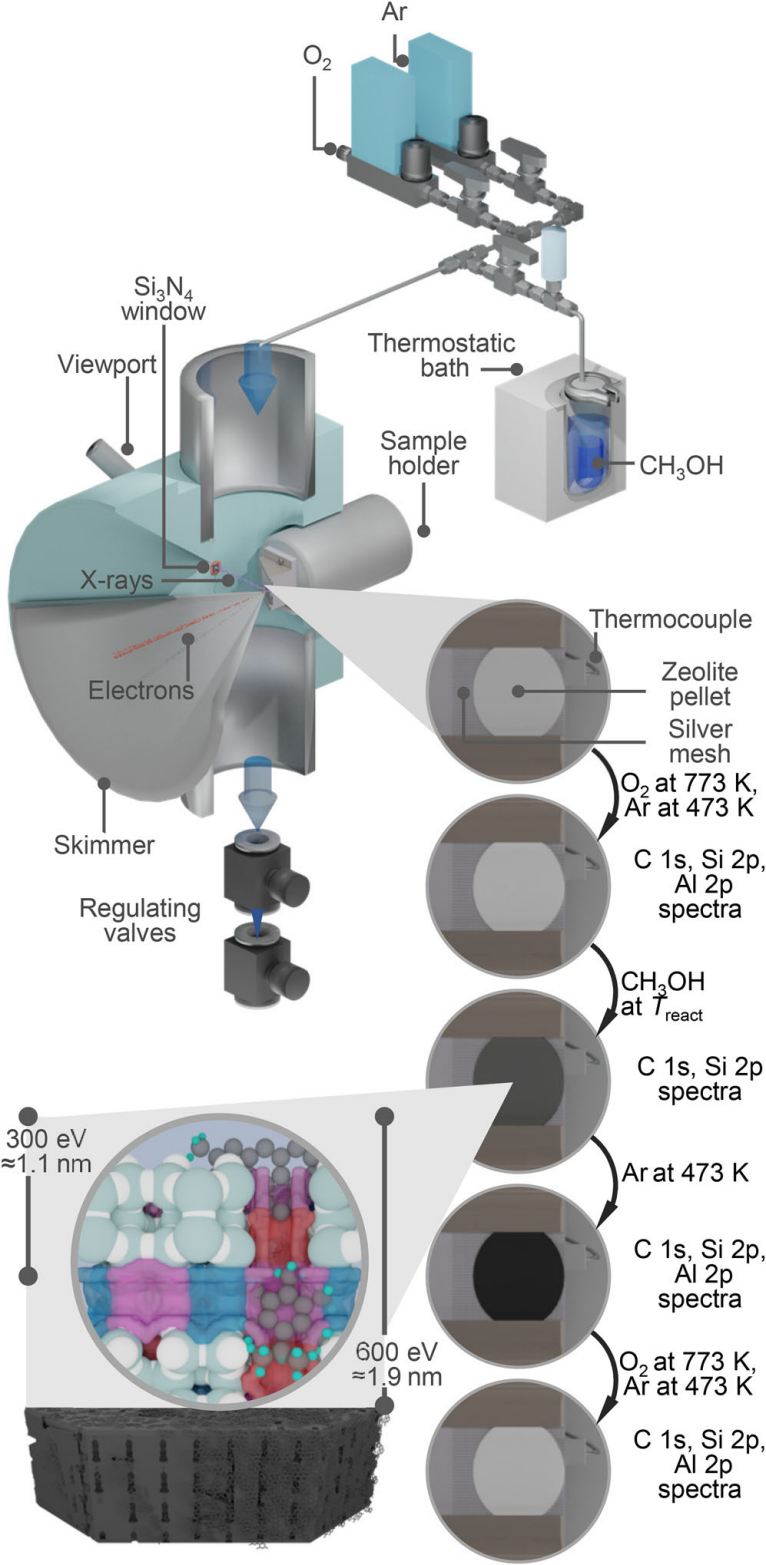
In situ XPS analysis of coking of ZSM‐5 catalysts. As shown by illustrations, after calcination at 773 K and cooling down under Ar flow, methanol was dosed over a catalyst pellet at 473 K, followed by a temperature increase to 773 K. Catalysts were cooled down under Ar flow and regenerated. C 1s, Si 2p, and Al 2p spectra were collected at selected kinetic energies to obtain nm‑scale surface information.

The kinetics of coke formation in the surface‐most region of the catalysts were elucidated by near‐ambient (*p* ≈ 1 mbar) pressure C 1s XPS analysis under in situ conditions (Scheme 1) [37, 38]. C 1s spectra were registered while flowing methanol feed over Z_15_ and Z_40_, previously calcined in the reaction chamber to eliminate adventitious carbon (Figure S4). The adjustment of the beam excitation energy enables the detection of emitted C 1s photoelectrons at constant kinetic energies (*E*_kin_ = 300 eV), corresponding to a mean escape depth in the nanometer range (*d ≈* 1.1 nm) [42]. Considering the unit cell size of ZSM‐5 (MFI framework) of *a* × *b* × *c* = 2.009 × 1.974 × 1.314 nm [43], the acquired C 1s spectra provide time‐resolved information on the accumulation of carbonaceous species at the crystal surface and the outer‐most micropore volume, where diffusion constraints are minimal (Scheme 1, inset), while avoiding potential artifacts associated with sample handling and exposure between the reactor and the analysis environment.

In situ acquisition and analysis of C 1s photoemission spectra present several challenges [37, 38, 44]. The most significant is sample charging, arising from the semiconducting nature of zeolites and common to virtually all realistic catalytic materials. Dispersing zeolite over a conductive silver mesh and maintaining a gas‐phase flow reduces these effects. Nonetheless, C 1s and Si 2p spectra display mostly a negative shift of *E*_kin_ that intensifies as the methanol conversion experiment proceeds, indicating charging (Figure S5). To overcome this, C 1s spectra were referenced to Si 2p spectra acquired in parallel (Figure S5). Si 2p binding energy was set to 103.5 eV, which is an average of commonly reported values for zeolites and silica‐like materials [45]. Importantly, Si 2p spectra acquired in parallel do not display peaks broadening, indicating that differential charging in the C 1s spectra is negligible (Figure S5).

**FIGURE 2:**
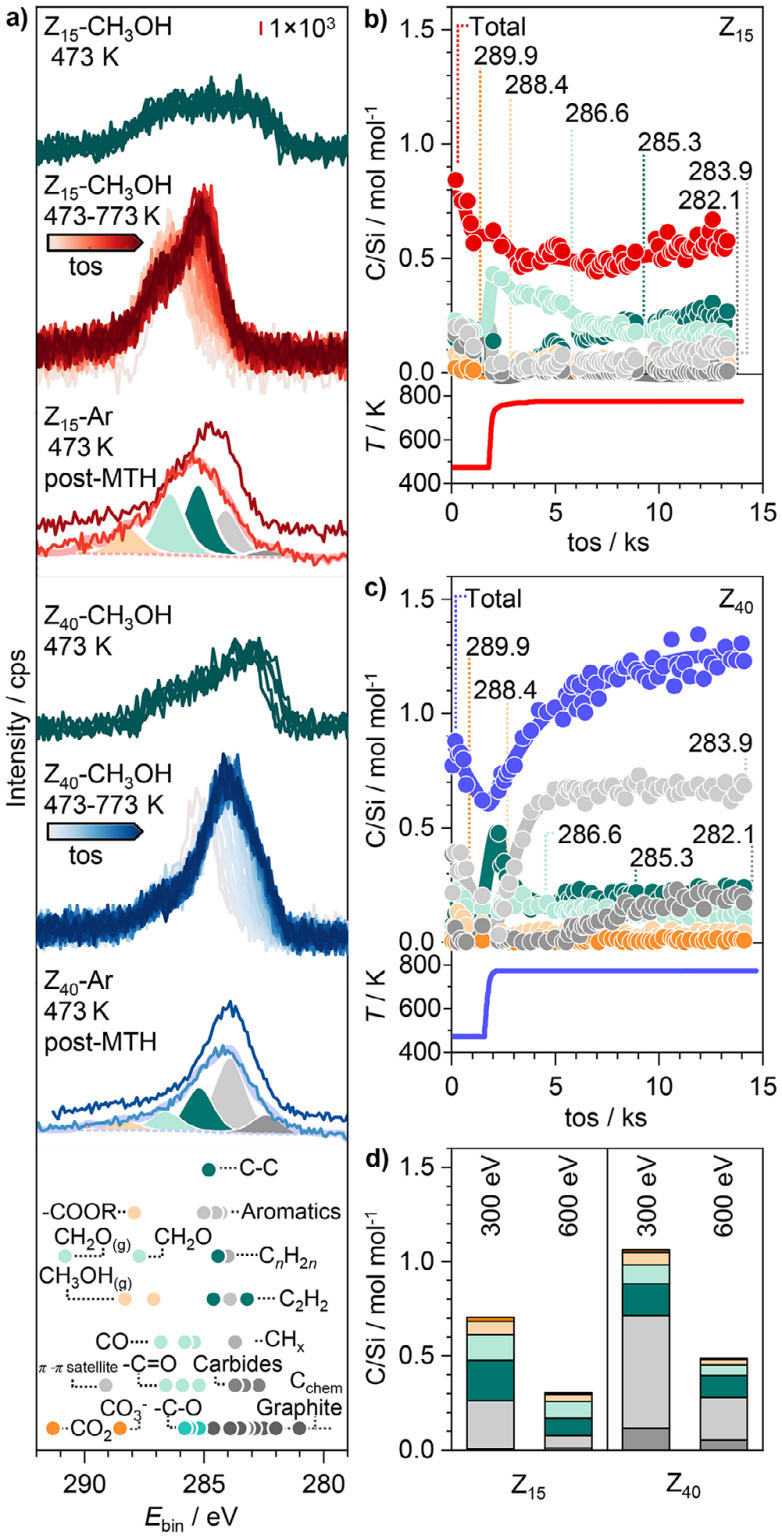
(a) In situ C 1s X‐ray photoemission spectra and (b, c) profiles of specific spectral components during methanol reaction (CH_3_OH) and (d) post‐reaction Ar exposure over Z_15_ and Z_40_ catalysts. Reference binding energy (*E*_bin_) ranges of specific carbon functionalities are shown below the spectra in (a). Spectral components in (b, c) were obtained after deconvolution as exemplified in (a). The temperature profiles during in situ methanol conversion experiments are displayed below the component profiles in (b, c). The C 1s photoelectrons were collected at a kinetic energy of *E*_kin_ = 300 eV and additionally at *E*_kin_ = 600 eV post‐reaction. Reaction conditions: CH_3_OH = 100 mol%, *T* = 273–773 K, and *P* = 1 mbar.

Internal referencing of the C 1s *E*_bin_ scale allows assigning peak components to carbonaceous compounds, based on the experimentally and theoretically‐assessed *E*_bin_ reported in the literature (Figure 2a, Table S3) [46, 47, 48, 49, 50, 51, 52, 53, 54, 55, 56, 57, 58, 59, 60, 61, 62, 63]. Although *E*_bin_ for specific functionalities exhibits some variability across studies, the C 1s reference scale can be divided into two regions. The lower range of *E*_bin_ ≈ 283.7–284.5 eV reflects carbon in saturated and unsaturated C–C bonds of adsorbed aliphatics and light aromatics. As unsaturation increases, leading to greater π‐bond delocalization, the *E*_bin_ values typically decrease. The range of *E*_bin_ ≈ 282–283 eV is associated with graphene‐like, highly dehydrogenated carbonaceous species [47, 48]. Although similar binding energies are observed for carbides [55], their formation under MTH conditions is thermodynamically highly unlikely [64]. The upper *E*_bin_ region reflects the oxygen‐containing carbon species, including surface alkoxy (C–O*, *E*_bin_ ≈ 284.2–285.8 eV), carbon monoxide and carbonyl compounds (C═O*, e.g., aldehydes, ketones, carboxylic acids, *E*_bin_ ≈ 285.2–287.8 eV), and gaseous species (CO_(g)_, e.g., methanol and formaldehyde, *E*_bin_ > 287.1 eV).

Following the in situ calcination and cooling down under argon, the reaction was initiated by dosing methanol at 473 K and *P* = 1 mbar (Figure 2a). At this temperature, DME formation is the dominant reaction, although the first C–C species may begin to form [12, 14]. C 1s spectra of Z_15_ and Z_40_ catalysts are relatively broad, spanning between ≈282 and 288 eV. The *E*_bin_ components centered at *E*_bin_ ≈ 288 eV, *E*_bin_ ≈ 285 eV, and *E*_bin_ ≈ 286 eV are ascribed to gaseous methanol/DME (C–O(H)_(g)_), surface adsorbed methanol/DME (C–O*) and surface carbonyl species (C═O*), respectively. Carbonyl species, such as formaldehyde, ketene, and acetyl, were identified as the prime intermediates participating in the C–C bond formation in the initial, low‐temperature phase of the MTH reaction [12]. The component at *E*_bin_ *≈* 284 eV can be associated with the surface methyl groups (SMS), and potentially first hydrocarbon species. The component at *E*_bin_ ≈ 283 eV may indicate the onset of aromatic HP formation.

The C/Si ratio exhibits initial fluctuations, which can be attributed to pressure and flow stabilization in the analysis chamber (Figure 2b,c, Figure S5). As the temperature increases from 473 to 773 K at a high heating rate, the total C 1s intensity initially decreases, indicating a desorption of carbon species from zeolite (Figure 2a–c), which also reduces the attenuation of the support signal (Figure S5). As illustrated by the difference spectra during heating up from 473 to 773 K, the C 1s intensity initially decreases rapidly in the spectral region *E*_bin_ ≈ 286–287 eV in Z_15_, and *E*_bin_ ≈ 285–286.5 eV in Z_40_ (Figure S6). These changes are accompanied by the progressive increase of the intensities in the spectral regions of *E*_bin_ ≈ 284–285.5 eV and *E*_bin_ ≈ 282–284.5 eV in Z_15_ and Z_40_, respectively. The intensity losses at *E*_bin_ ≈ 285 eV and *E*_bin_ ≈ 287 eV suggest the consumption of C–O* and C═O* compounds, respectively. Meanwhile, the growing intensity at *E*_bin_ ≈ 284 eV suggests the formation of aliphatics and light aromatics, containing sp^3^ and sp^2^ hybridized carbons.

Deconvolution of C 1s spectra provides insights into temporal changes of carbonaceous species, enabling also to estimate the rates of their formation (Figure 2b,c and Figure S7). In both catalysts, an initial fast phase and a later slow phase of coke formation are observed. The C/Si ratio is higher for Z_40_ and increases more markedly over time, whereas in Z_15_ it rises only moderately, indicating more pronounced surface coking on the former catalyst. In Z_15_, the component at *E*_bin_ ≈ 286.6 eV, attributed to oxygen‐containing species, is initially prominent and decreases at the highest rate (≈3 × 10^−5^ s^−1^) in the initial phase (Figure S7). This coincides with the growth of hydrocarbon‐related components at *E*_bin_ ≈ 285.3 (≈4 × 10^−5^ s^−1^) and 283.9 eV (≈2 × 10^−5^ s^−1^), with former increasing at a higher rate in both reaction phases. The temporal C 1s spectra profile of Z_15_ suggests that coke growth arises from the conversion of oxygenates, consistent with the depletion of methoxy species, with potential involvement of carbonyl compounds during the initial phase of the MTH reaction [12, 14]. The higher prevalence of oxygenates in Z_15_ can be associated with higher BAS concentration, which promotes the formation of formaldehyde from methanol and its conversion to unsaturated hydrocarbons [18, 26].

In Z_40_, during the initial phase, the oxygen‐containing component at *E*_bin_ ≈ 286.6 eV decreases at a comparatively lower rate (≈1 × 10^−5^ s^−1^) than in Z_15_ (Figure S7). Contrastingly, the hydrocarbon component at *E*_bin_ ≈ 285.3 eV decays at a high rate (≈1.3 × 10^−4^ s^−1^), while another hydrocarbon component at *E*_bin_ ≈ 283.9 eV increases at an even higher rate (≈2.4 × 10^−4^ s^−1^). However, in the late phase, both change at a low rate (in the range of ≈1 × 10^−6^ s^−1^). The most striking difference in Z_40_ compared to Z_15_ is the emergence and growth of the component at *E*_bin_ ≈ 282.1 eV, which can be associated with highly dehydrogenated, graphite‐like coke species [47, 48]. Notably, in the later reaction phase, this component increases steadily at the highest rate (≈2.3 × 10^−5^ s^−1^), thus contributing mostly to the increase of carbon content. This observation aligns with the higher graphitic character of coke revealed by Raman and EELS analyses of the deactivated catalysts (Figure 1d–h). Additionally, while lighter hydrocarbon and oxygenate components dominate the Z_15_ spectra, hydrocarbon‐derived species prevail in Z_40_, with their kinetics indicating graphitic coke formation likely via secondary conversion of lighter hydrocarbons.

**SCHEME 2:**
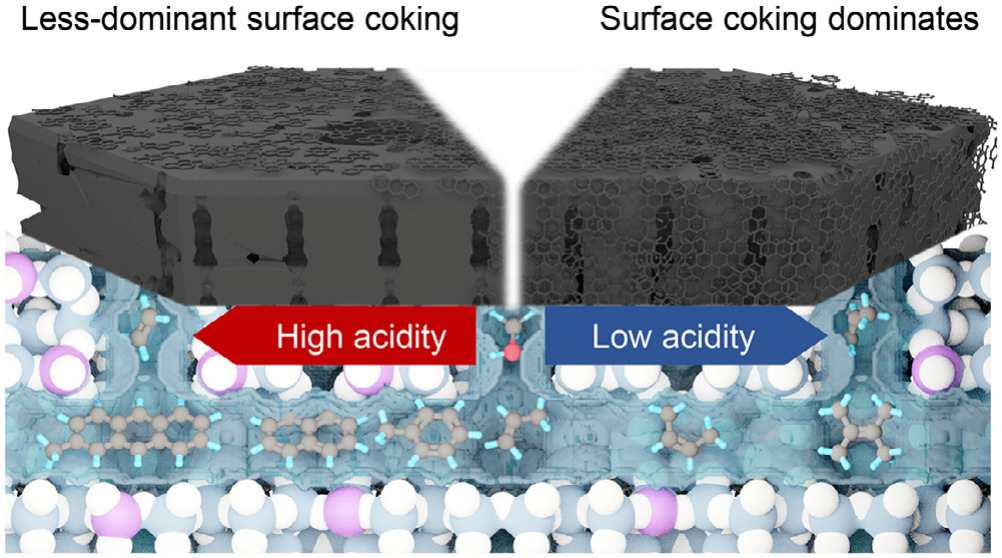
Differences in surface coking of ZSM‑5 catalysts as a function of acidity.

After a specific time, the methanol feed was replaced with an argon flow to remove weakly adsorbed species. The C 1s spectra acquired at *hν* = 595 eV after cooling down to 473 K show similar profiles, with only minor variations in C/Si ratios compared to those at the final reaction point (Figure 2d). Deactivated catalysts show slightly higher surface Si/Al ratios than the calcined or regenerated ones, particularly Z_40_, likely due to more pronounced surface coking (Figure S8, Table S4). Overall, despite slower deactivation kinetics, the Z_40_ catalyst exhibits higher surface C/Si ratios than Z_15_ and a significant fraction of the low‑energy component (*E*_bin_ ≈ 282.1 eV), indicating more prevalent surface coking and the formation of graphitic‑like coke (Scheme 2).

## 3 Conclusion

In summary, in situ XPS offers exceptional resolving power for elucidating the coking mechanism, which is a ubiquitous cause of deactivation in zeolite‐catalyzed processes. It reveals stronger surface coking on the less acidic catalyst and a larger low‐binding‐energy C 1s contribution, indicating higher graphitization. These coke‐evolution trends observed from in situ XPS are consistent with complementary spectroscopic measurements at mbar pressures, as well as with kinetic, lattice‐expansion, and coke‐analysis experiments conducted at bar‐level pressures. This agreement indicates that the differences in coking behavior between low‐ and high‐acidity ZSM‐5 catalysts persist across a broad pressure range spanning three orders of magnitude. Thus, acid‐site concentration governs both coke‐formation kinetics and the preferential region of coke deposition. These insights show that, beyond optimizing micropore topology and acidity, tailoring catalyst surface properties is essential for improving MTH catalyst performance. The presented methodology provides an effective tool for elucidating the mechanisms of the surface catalyst coking as a function of zeolite structure and reaction conditions, also in transformations beyond the MTH conversion.

## Author Contributions

**Luca Artiglia**: methodology, formal analysis, writing – review and editing, validation. **Hannes Frey**: formal analysis, data curation, writing – review and editing. **Loïc Bénariac‐Doumal**: writing – review and editing, formal analysis, data curation. **Sung Sik Lee**: formal analysis, data curation, writing – review and editing.**Jeroen A. van Bokhoven**: funding acquisition, writing – review and editing, resources, supervision. **Przemyslaw Rzepka**: writing – review and editing, formal analysis, data curation. **Vladimir Paunović**: conceptualization, investigation, writing – original draft, methodology, visualization, formal analysis, data curation, project administration.

## Conflicts of Interest

The authors declare no conflicts of interest.

## Supporting information




**Supporting file 1**: anie72899‐sup‐0001‐SuppMat.pdf.

## Data Availability

The data that support the findings of this study are available from the corresponding author upon reasonable request.
