# Supplementary material for: Spatial Evolution of Coke in ZSM‐5 Catalysts During Methanol‐to‐Hydrocarbons Conversion Revealed by In Situ X‐Ray Photoelectron Spectroscopy
Source: Angew Chem Int Ed Engl. 2026 Jun 13;65(31):e7876229. doi: 10.1002/anie.7876229 (PMC13411610; doi:10.1002/anie.7876229)
Supplement: Supplementary file 1 — Supporting file 1: anie72899‐sup‐0001‐SuppMat.pdf. [file ANIE-65-e7876229-s001.pdf]

Supporting Information

Spatial Evolution of Coke in ZSM-5 Catalysts during Methanol-to-Hydrocarbons Conversion Revealed by In Situ X-ray Photoelectron Spectroscopy

Luca Artiglia,[a] Hannes Frey,[b] Loïc Bénariac-Doumal,[c] Sung Sik Lee,[d] Przemyslaw Rzepka,[c] Jeroen A. van Bokhoven,[a,b] and Vladimir Paunović [a]**

[a] PSI Center for Energy and Environmental Sciences, Paul Scherrer Institute, Forschungsstrasse 111, 5232 Villigen, Switzerland.

[b] Institute for Chemical and Bioengineering, ETH Zurich, Vladimir-Prelog-Weg 1-5, 8093 Zurich, Switzerland.

[c] J. Heyrovsky Institute of Physical Chemistry, Czech Academy of Sciences, Prague 8, 182 23, Czech Republic.

[d] Scientific Center for Optical and Electron Microscopy (ScopeM), Otto-Stern-Weg 3, 8093 Zurich, Switzerland.

*E-mails: jeroen.vanbokhoven@chem.ethz.ch

vladimir.paunovic@psi.ch

S1. SUPPORTING EXPERIMENTAL INFORMATION

S1.1. Catalysts. ZSM-5 zeolites with nominal Si/Al ratios of 15 (CBV 3024E) and 40 (CBV 8014) were obtained from Zeolyst in ammonium form and converted to a proton form by calcination under oxygen (PanGas, 5.0) flow (F_T = 100 cm^3_{STP} min^{-1}) at 823 K for 5h, using a heating rate of 5 K min^{-1}.

S1.2. Catalyst Characterization. Powder X-ray diffraction (XRD) was measured ex situ in Bragg-Brentano geometry using a PANalytical X'Pert PRO-MPD diffractometer. The data was recorded with Cu Kα radiation (λ = 0.154 nm) with a 2θ range of 5–70° with an angular step size of 0.05° and a counting time of 1.5 s per step. Pawley refinements were performed using TOPAS 7 software. [1] The lattice parameters were extracted from the positions of the Bragg reflections corresponding to the orthorhombic *Pnma* space group of the ZSM-5 zeolite (MFI topology). The background was modeled using a Chebyshev polynomial, and the peak shapes were described by a pseudo-Voigt function. The same axial divergence model was fixed for all diffractograms, while the zero-shift parameter was allowed to vary.

Nitrogen sorption was measured at 77 K on a BELSORP MAX X Surface Area and Porosity Analyzer. Prior to the measurement, the zeolite sample (*ca*. 0.1 g) was degassed for 12 h at 573 K.

The morphology as well as silicon and aluminum content of the zeolite catalysts were analyzed *via* scanning electron microscopy (SEM), coupled with energy dispersive X-ray spectroscopy (EDS). The instrument, a Hitachi SU5000, is equipped with both two SE-detectors and two EDS detectors. The accelerating voltage used was 20 kV.

Scanning Transmission Electron Microscopy (STEM), Electron Energy Loss Spectroscopy (EELS) and EDS were performed using a JEOL Grand ARM Cs corrected electron microscope equipped with a cold field emission gun (ΔE ≈ 0.35 eV), SDD EDX detectors with a total area of 100 mm^2. The microscope was operated at 300 keV, EELS was recorded using a Model 965 GIF Quantum ER EELS Spectrometer. To prevent additional carbon deposition during imaging or sample preparation, the samples were drop casted on a Protochips® Fusion Select heating chip with a perforated SiN_x membrane using ultrapure water. The chips were then tried overnight at room temperature and under vacuum. Prior to imaging and spectra acquisition, the chip was heated in the ultrahigh-vacuum chamber to 673 K at 10 K min^{-1} to desorb weakly adsorbed species while remaining well below the reaction temperature to limit coke

transformation, with both zeolites undergoing the same treatment. mEDS Spectral analysis was done using the hyperspy python package (hyperspy.org), the used code can be made available by request. EELS Spectra were processed using Digital Micrograph (Gatan, USA) by aligning the recorded high loss to the zero loss and applying a power law background removal as well as FFT filtering for denoising.

Fourier transform infrared spectroscopy (FTIR) measurements were performed on Excalibur spectrometer equipped with a DTGS detector. A self-supported discs (*ca*. 17 mg, 1.5 cm^2) of zeolites were placed in a quartz FTIR cell and degassed at 723 K and pressure better than 5×10^{-6} bar for 3 h. The FTIR spectra were collected after cooling down to room temperature. Pyridine (abcr, ≥99.5%) vapor (2.5×10^{-4} bar) or 2,6-di-tert-butylpyridine (Apollo Scientific, ≥97%) vapor (2×10^{-5} bar) was then dosed. The amount of adsorbed probe molecule was analyzed by recording the spectra after evacuating the sample at 573 K for pyridine and 323 K for 2,6-di-tert-butylpyridine at pressure better than 5×10^{-6} bar for 1 h. All FTIR spectra were collected by accumulating 128 scans in the range 650-4000 cm^{-1} with a nominal resolution of 4 cm^{-1}. Concentration of BAS and LAS and surface BAS and LAS was calculated from the pyridine and 2,6-di-tert-butylpyridine adsorption spectra, respectively, using previously reported excitation coefficients.[2,3]

Solid-state ^{27}Al magic-angle spinning nuclear magnetic resonance (^{27}Al MAS NMR) spectroscopy measurements were performed on a Bruker Avance IIIHD 700 spectrometer equipped with a 3.2 mm Double Resonance MAS probe head. The samples were hydrated over an aqueous NH_4NO_3 solution (1 M) for 48 h and then filled into 3.2 mm zirconia rotors. The spectra were recorded at a ^{27}Al resonance frequency of 182.4 MHz using a single-pulse excitation with SPINAL64 decoupling on the ^{1}H channel during acquisition. The excitation pulse was set to 1.5 ms (corresponding to flip angle of *ca*. π/6). A recycle delay of 1 s, a sweep width of 979 ppm, an acquisition time of 0.034 s and 4096 scans were used. The spinning rate was set to 16.00 kHz. The spectra were normalized to the sample weight. Solid state ^{29}Si magic angle spinning nuclear magnetic spectroscopy ^{29}Si MAS NMR spectra were measured on a Bruker Avance III HD 400 spectrometer equipped with a Double Resonance MAS 4 mm probe head at a resonance frequency of 79.5 MHz using single-pulse excitation sequence with a pulse duration of 1.7 ms (corresponding to a flip angle of *ca*. π/6). A recycle delay of 4 s, a sweep width of 349 ppm, an acquisition time of 0.028 s and 10240 accumulations were used. The spinning rate was set to 5 kHz. In both ^{27}Al and ^{29}Si MAS NMR experiments, the magnetic field and therefore the ppm axis were

calibrated using the ^{13}C MAS NMR spectrum of adamantane (at 38.52 ppm) as an external secondary standard.

Thermogravimetric analysis (TGA) was performed on a Netzsch STA 449 C Jupiter Thermo microbalance analyzer. The samples were first heated to 473 K and then to 1073 K under a flow of synthetic air (PanGas, 5.0, F_{O2} = 20 cm^3_{STP} min^{-1}, PanGas, 5.0, F_{N2} = 80 cm^3_{STP} min^{-1}) using a heating rate of 5 K min^{-1} and 10 K min^{-1} respectively with dwell times of 1 and 0.5 h. Any weight loss below the temperature of 573 K was attributed to the evaporation of water, and the coke content is determined from the weight loss above 573 K.

The diffusion measurements were performed by measuring the release of toluene by a TGA. The crucible was filled with *ca*. 55 mg of catalyst. The sample was heated to 873 K for 2 h with a heating rate of 10 K min^{-1} to remove any moisture, followed by cooling down to 333 K, both under nitrogen flow (F_{N2} = 200 cm^3_{STP} min^{-1}). Toluene (Sigma Aldrich, >99.5%) was loaded by passing nitrogen (F_{N2} = 200 cm^3_{STP} min^{-1}) through a bubbler filled with this hydrocarbon and placed in an ice-cooled bath. After the adsorption curve achieved saturation, the bubbler feed was stopped, and nitrogen was passed through to desorb toluene for *ca*. 2 ks. The effective diffusion constant, $D_{eff}R^{-2}$, was determined by non-linear regression of the desorption curve using a model derived from the second Fick's law of diffusion for a spherical geometry formulated by **Equation S1**, in which w is the uptake of toluene at time t, w_0 is the uptake at saturation.[4] The fitting was implemented in MATLAB.

$$\frac{w}{w_0} = 1 - \frac{6}{\pi^2}\sum_{1}^{\infty}\frac{1}{n^2}e^{-n^2\pi^2 D_{eff} R^{-2} t}, \quad - \qquad \textbf{S1}$$

Raman spectra were acquired by using a Horiba LabRAM HR Evolution instrument in a configuration that exploits 325 nm laser (source power 25 mW), 1800 mm^{-1} grating, 40x objective lens (Thorlabs LMU-40X UVB), edge and holographic notch filters, and Horiba Synapse CCD detector. The samples were prepared in the form of self-supported discs, and the spectra were acquired in the Raman shift range of 300-1700 cm^{-1} with 30 s acquisition time. The absence of laser-induced sample damage was ensured by visual inspection.

S.1.3. Catalyst Testing. The MTH conversion reaction tests were carried out in an automated homemade continuous-flow fixed-bed reactor setup, described previously.[5] Prior to reaction, the

catalyst was heated under argon flow (F_{Ar} = 300 cm^3_{STP} min^{-1}) to 823 K (heating rate of 10 K min^{-1}), then activated under oxygen (F_{O2} = 100 cm^3_{STP}). The reaction tests were conducted using methanol (CH_3OH Sigma Aldrich, HPLC grade, >99.9%,), concentration of c_{CH3OH} = 19 mol%, weight-hourly space-velocity of WHSV = 72 g_{CH3OH} g_{cat} h^{-1}at T = 773 K and total pressure of P = 1.8 bar.

The inlet and outlet reactor feeds were analyzed by a gas chromatograph (Agilent 6890) equipped with a sampling valve, PLOT-Q column, and a flame-ionization detector. The conversion (X), selectivity to hydrocarbon products C_xH_y (S_{CxHy}), hydrogen transfer index (HTI), and cumulative turnover (CT) were calculated according to **Equations S2-5**, in which $n_{i,\text{ in}}$ and $n_{i,\text{ out}}$ are the molar flows of compound i at reactor inlet and outlet, x is the number of carbon, y is the number of hydrogen or deuterium atoms, p is the total number of detectable products, and $WHSV$ is the weight hourly space velocity. The relative carbon balance error was in the range of ±5%.

$$X = (1 - \frac{n_{\text{CH}_3\text{OH, out}} + n_{\text{DME, out}}}{n_{\text{CH}_3\text{OH, in}} + n_{\text{DME, in}}}) \times 100,\ \% \qquad \textbf{S2}$$

$$S_{\text{C}_x\text{H}_y} = \frac{x n_{\text{C}_x\text{H(D)}_y}}{\sum_1^p x n_{\text{C}_x\text{H(D)}_y}} \times 100,\ \% \qquad \textbf{S3}$$

$$\text{HTI} = \frac{n_{\text{C}_4\text{H(D)}_{10}}}{n_{\text{C}_4\text{H(D)}_{10}} + n_{\text{C}_4\text{H(D)}_8}} \times 100,\ \% \qquad \textbf{S4}$$

$$\text{CT} = \text{WHSV} \int X dt,\ \text{g}_{\text{CH}_3\text{OH}}\ \text{g}_{\text{cat}}^{-1} \qquad \textbf{S5}$$

S.1.4. *Operando* UV-Vis and *in situ* FTIR experiments*.* *In situ* FTIR studies of MTH reaction were performed using the same home-built cell and FTIR instrument. The samples (*ca*. 17 mg, *ca*. 1.5 cm^2) were first degassed in at 773 K. The pellet was then cooled and the spectra of the pre-treated material were collected. Next, 1.2 mbar of methanol vapors were introduced into the cell, and the pellet was allowed to react for 1 min. The reaction was quenched by transferring the pellet to an intermediate zone, located between the transmission and heated zones, while the FTIR spectra of the gas phase were collected. The pellet was then moved to the transmission zone of the cell, and FTIR spectra were recorded. Afterwards, the pellet was returned to the heated zone, and the procedure repeated for specified time, without introducing additional methanol. Optical adsorption was measured in the range

of 400 to 4000 cm^{-1} with a resolution of 4 cm^{-1} and 128 total scans. Spectra of surface species were obtained by subtracting the post-reaction gas-phase spectra from the spectra collected with the pellet inside the transmission zone. *Operando* DR-UV-vis analysis of the reaction intermediates retained by the zeolite catalysts was performed in a home-built quartz cell, connected to identical feeding and analytics system used for the fixed-bed microreactor tests. The MTH catalytic tests were performed using a feed of CH_3OH:Ar = 0.5:99.5 mol%, $WHSV$ = 4.5g_{CH3OH} g_{cat}^{-1} h^{-1}, T = 773 K, and P = 1.2 bar. Background reference spectra were collected before the reaction mixture was introduced. Thereafter, spectra were collected continuously (v_{scan} = 0.5 Hz) using an Ocean Optics DH-2000-BAL deuterium/halogen light source, a 200-1100 nm Ocean Optics six-around-one reflection probe, and an Ocean Optics Maya 2000-Pro UV-vis spectrometer. In registering the DR-UV-vis spectra, the spectra of the bare, calcined zeolite recorded just before the reaction mixture was admitted were taken as a refence, and the subsequent spectra were collected without any additional background subtraction.

S1.5. *In situ* XPS Experiments*.* *In situ* XPS studies of MTH reaction were performed at the X07DB In Situ Spectroscopy beamline at the Swiss Light Source (SLS), Paul Scherrer Institute, using the solid–gas interface endstation enabling precise gas dosing under controlled flow conditions.[6–8] Zeolite powder was pressed into a pellet (*ca*. 1.5 cm^2) over a silver gauze, which was added to reduce the charging effects. The pellet was loaded on a holder, which enables temperature control by combining laser heating and a temperature sensor (Pt100) in direct contact with the catalyst. After loading the pellet in the analysis chamber, it was pretreated by heating up under oxygen flow (5 cm^3_{STP} min^{-1}) at P = 1 mbar to T = 773 K, where it was kept for 30 mins to remove the adventitious carbon from the catalyst, as confirmed by recording the survey and C 1s spectra (**Figure S4**). Thereafter, the oxygen flow was replaced with argon (3 cm^3_{STP} min^{-1}) and pellet was cooled down to 473 K. Methanol was then fed from a thermostatic reservoir at a flow rate of F_{CH3OH} ≈ 1 cm^3_{STP} min^{-1} and P = 1 mbar. The temperature was then rapidly increased (heating rate 1.1 K s^{-1}) to T = 773 K, at which the MTH reaction takes place. During the methanol feeding, C 1s and Si 2p spectra were continuously collected by using linearly-polarized light and at a photon energy of hv = 595 eV. This results in a kinetic energy of C 1*s* photoelectrons of E_{kin} = 300 eV. The mean escape depth of d ≈ 1.1 nm was calculated for this kinetic energy at an electron emission angle of θ = 30°, using an inelastic mean free path (λ) obtained from the NIST Electron Inelastic Mean Free Path Database via the Gries equation and assuming that the properties of the zeolites correspond to those of SiO_2 (1 g cm^{-3}, **Equation S6**).[9] Typically, five C 1s and

one Si 2p sweeps were taken in an individual scan and averaged over a period of 144 s (Z_{15}) and 130 s (Z_{40}) to obtain an average time point (**Figure 2**).

Pre- and post-reaction spectra were collected under Ar flow at 473 K by adjusting the excitation energies for C 1s (595, 895 eV), Si 2p (415, 720 eV), and Al 2p (390, 690 eV) to register the photoelectrons at kinetic energies of 300 eV and 600 eV, which corresponds to MED ≈ 1.1 and 1.9 nm, respectively. The intensity of the spectra and thus the integrated areas were normalized (I_{norm}) to the total number of scans (n_{scans}), the beam photocurrent ($I_{h\nu}$), the fraction of first order light (f_1), the photoionization cross section (σ), and the influence of asymmetry of the respective elemental orbital (β), with all parameters calculated at the corresponding energy ($h\nu$) at which spectra (I) were registered (**Equation S7**). After Shirley background subtraction ($I_{Shirley}$) and referencing to Si 2p energy of 103.5 eV,[10] the spectra were numerically integrated and deconvoluted by using CasaXPS software. Surface C/Si and Si/Al elemental ratios (X_1/X_2) were calculated by dividing the numerical integrals obtained from the respective normalized spectra recorded at specific kinetic energy of photoelectrons (**Equation S8**).

$$d = \lambda \times \cos(\theta), \text{ nm} \qquad \textbf{S6}$$

$$I_{\mathrm{norm}}(h\nu) = \frac{I(h\nu)}{n_{\mathrm{scans}} \times \frac{I_{ph}(h\nu)\times 3.76}{h\nu} \times f_1(h\nu) \times \frac{\sigma(h\nu)}{4\pi} \times (1+\beta(h\nu))}, \text{ -} \qquad \textbf{S7}$$

$$X_1 / X_2 = \frac{\int \left(I_{\mathrm{norm}}(h\nu) - I_{\mathrm{Shirley}}(h\nu) \right)\Big|_{X_1,\, E_{\mathrm{kin}}}}{\int \left(I_{\mathrm{norm}}(h\nu) - I_{\mathrm{Shirley}}(h\nu) \right)\Big|_{X_2,\, E_{\mathrm{kin}}}}, \text{ mol mol}^{-1} \qquad \textbf{S8}$$

Table S1. Composition, textural, and acid properties of fresh ZSM-5 catalysts.

Catalyst	Si/Al$_{bulk}$[a] / mol mol^{-1}	Si/Al$_{surf}$[b] / mol mol^{-1}	S_{meso}[c] / m^2 g^{-1}	S_{BET}[d] / m^2 g^{-1}	V_{micro}[c] / cm^3 g^{-1}	V_{total}[d] / cm^3 g^{-1}	$c_{BAS, bulk}$[e] / μmol g^{-1}	$c_{LAS, bulk}$[e] / μmol g^{-1}	$c_{BAS, surf}$[f] / μmol g^{-1}	$D_{eff}R^{-2}$[g] / s^{-1}
Z_{15}	22	19	24	323	0.16	0.22	391	172	9	2.2 ×10^{-6}
Z_{40}	39	48	55	362	0.16	0.22	217	69	3	2.2 ×10^{-6}

[a] ^{29}Si MAS NMR. [b] Si 2p and Al 2p X-ray photoemission spectra at E_{kin} = 300 eV. [c] *t*-plot and [d] BET analysis of N_2 sorption isotherms. [d] From N_2 adsorbed volume at p/p^0 = 0.99. FTIR analysis of [e] pyridine adsorption at 573 K and 2,6-di-tert-butylpyridine at 323 K. [f] Toluene desorption at 573 K.

Table S2. Unit cell parameters of ZSM-5 catalysts in their fresh and deactivated forms.[a]

X / %	***a* /** Å	*σ**a*** / Å	***b* /** Å	*σ**b*** / Å	***c* /** Å	*σ**c*** / Å	*a-b* / Å	*σ(a-b)* / Å
				Z_{15}				
fresh	20.23131	0.0068	19.96524	0.00827	13.42381	0.00818	0.26607	0.010707
100	20.22913	0.00535	19.97354	0.00424	13.43404	0.00342	0.25559	0.006826
99	20.19487	0.00502	19.96619	0.00424	13.42999	0.00382	0.22868	0.006571
86	20.16496	0.00508	19.98279	0.00613	13.42906	0.00715	0.18217	0.007961
29	20.23256	0.00894	20.03391	0.00516	13.43457	0.00371	0.19865	0.010322
				Z_{40}				
fresh	20.22462	0.01164	19.93565	0.01015	13.41214	0.00788	0.28897	0.015444
100	20.23629	0.00956	19.9612	0.0104	13.40336	0.01439	0.27509	0.014126
100	20.21488	0.00715	19.96106	0.00584	13.41475	0.00451	0.25382	0.009232
98	20.19951	0.00779	19.95993	0.01496	13.40325	0.01109	0.23958	0.016867
78	20.24053	0.01073	20.00569	0.00769	13.40536	0.00525	0.23484	0.013201
25	20.25376	0.01052	20.03317	0.01528	13.43685	0.01192	0.22059	0.018551

[a] The lattice parameters were obtained from Pawley refinements.

Table S3. Binding energies of carbon functionalities in the C 1s spectra.

E_{bin} / eV	Assignment[a]
281-282	Highly-dehydrogenated carbon species on Al_2O_3[11]
282	Aluminum carbide[12]
282	Metal carbides[13]
282.5	Chemisorbed carbon[14]
282.7	Metal carbides[15]
282.9	Chemisorbed carbon[14]
283.2	H-poor sp or graphitic carbon[15]
283.2	di-σ bounded ethene[16]
283.3	Carbidic carbon[14]
283.4	Adsorbed carbon[13]
283.5	Surface graphite[17]
283.7	Adsorbed CH_x species[18]
283.7	Carbidic C[14]
283.9	Graphene[12]
283.9	Acetylene[19]
284	Carbon sp^2 species[20]
284.3	Naphthalene[14]
284.4	Carbon species with C=C bonds[21]
284.5	Benzene[19]
284.6	Graphitic carbon[15]
284.6	Propene[17]
284.7	Carbon species with C-C bonds[21]
284.8	Carbon sp^3 species[20]
285	Benzene[19]
285.2	Adsorbed CH_3O species[18]
285.2	Carbon species with C-H, C-OR-OH, R-O-R, Ar-OH bonds[21]
285.2	Carbon species with C=O bonds[22]
285.4	Adsorbed CO[12]
285.6	Adsorbed CH_xO, -OH, CH_3O,CH_2O, HCO, CO species[18]
285.8	Carbon species with C−O(H) bonds[20]
285.8	CO[12]
285.9	Carbon species with C=C-O bonds, furane, ketoenol[21]
286.6	Carbon species with C=O bonds[21]
286.8	Adsorbed CO[20]
286.8	Adsorbed CO (top)[12]
287.1	Gaseous methanol[20]
287.7	Formaldehyde[18]
287.9	Carbon species with COOH, COOR functionalities[21]
288.3	Gaseous methanol[18]
288.5	Surface carbonates[21]
289.1	π-π satelite transition[21]
290.8	Gaseous formaldehyde[18]
291.3	Adsorbed carbon dioxide[12]
294	Gaseous carbon dioxide[18]

[a]Reference.

Table S4. Surface Si/Al and C/Si ratios of the ZSM-5 catalysts, along with the corresponding parameters used for their calculation from the *in situ* XPS experiments.

Catalyst	E_{kin} / eV	Spectra	I / n_{scans} / cps	$h\nu$ / eV	I_{ph} / μA	f_1 / -	σ / -	β / -	Si/Al / mol mol^{-1}	C/Si / mol mol^{-1}
					Z_{15}					
Calcined	300	Al 2p	2.75×10^3	385	1.40	0.81	0.43	1.46	19	b.d.[a]
		Si 2p	3.57×10^4	415	1.94	0.69	0.49	1.47		
	600	Al 2p	8.79×10^3	690	9.85	0.82	0.08	1.29	14	b.d.
		Si 2p	9.94×10^4	720	10.13	0.89	0.10	1.33		
Post-MTH	300	Al 2p	2.39×10^3	385	1.57	0.81	0.43	1.46	21	0.78
		Si 2p	3.50×10^4	415	2.14	0.69	0.49	1.47		
		C 1s	2.41×10^4	595	7.60	0.64	0.17	2.00		
	600	Al 2p	7.91×10^3	690	10.28	0.82	0.08	1.29	15	0.35
		Si 2p	9.58×10^4	720	10.60	0.89	0.10	1.33		
		C 1s	2.38×10^4	905	14.12	1.00	0.06	2.00		
Regenerated	300	Al 2p	3.06×10^3	385	1.55	0.81	0.43	1.46	18	b.d.
		Si 2p	3.92×10^4	415	2.21	0.69	0.49	1.47		
	600	Al 2p	9.07×10^3	690	10.30	0.82	0.08	1.29	15	b.d.
		Si 2p	1.10×10^3	720	10.56	0.89	0.10	1.33		
					Z_{40}					
Calcined	300	Al 2p	1.01×10^3	385	1.42	0.81	0.43	1.46	48	b.d.
		Si 2p	3.31×10^4	415	1.95	0.69	0.49	1.47		
	600	Al 2p	2.30×10^3	690	9.71	0.82	0.08	1.29	47	b.d.
		Si 2p	8.82×10^4	720	10.24	0.89	0.10	1.33		
Post-MTH	300	Al 2p	7.85×10^4	385	1.55	0.81	0.43	1.46	56	1.00
		Si 2p	2.90×10^4	415	2.03	0.69	0.49	1.47		
		C 1s	2.62×10^4	595	7.43	0.64	0.17	2.00		
	600	Al 2p	2.00×10^3	690	10.15	0.82	0.08	1.29	53	0.46
		Si 2p	8.50×10^4	720	10.50	0.89	0.10	1.33		
		C 1s	2.27×10^4	905	11.41	1.00	0.06	2.00		
Regenerated	300	Al 2p	1.12×10^3	385	1.55	0.81	0.43	1.46	44	b.d.
		Si 2p	3.23×10^4	415	2.04	0.69	0.49	1.47		
	600	Al 2p	2.53×10^3	690	10.10	0.82	0.08	1.29	44	b.d.
		Si 2p	9.00×10^4	720	10.43	0.89	0.10	1.33		

[a]Below detection.

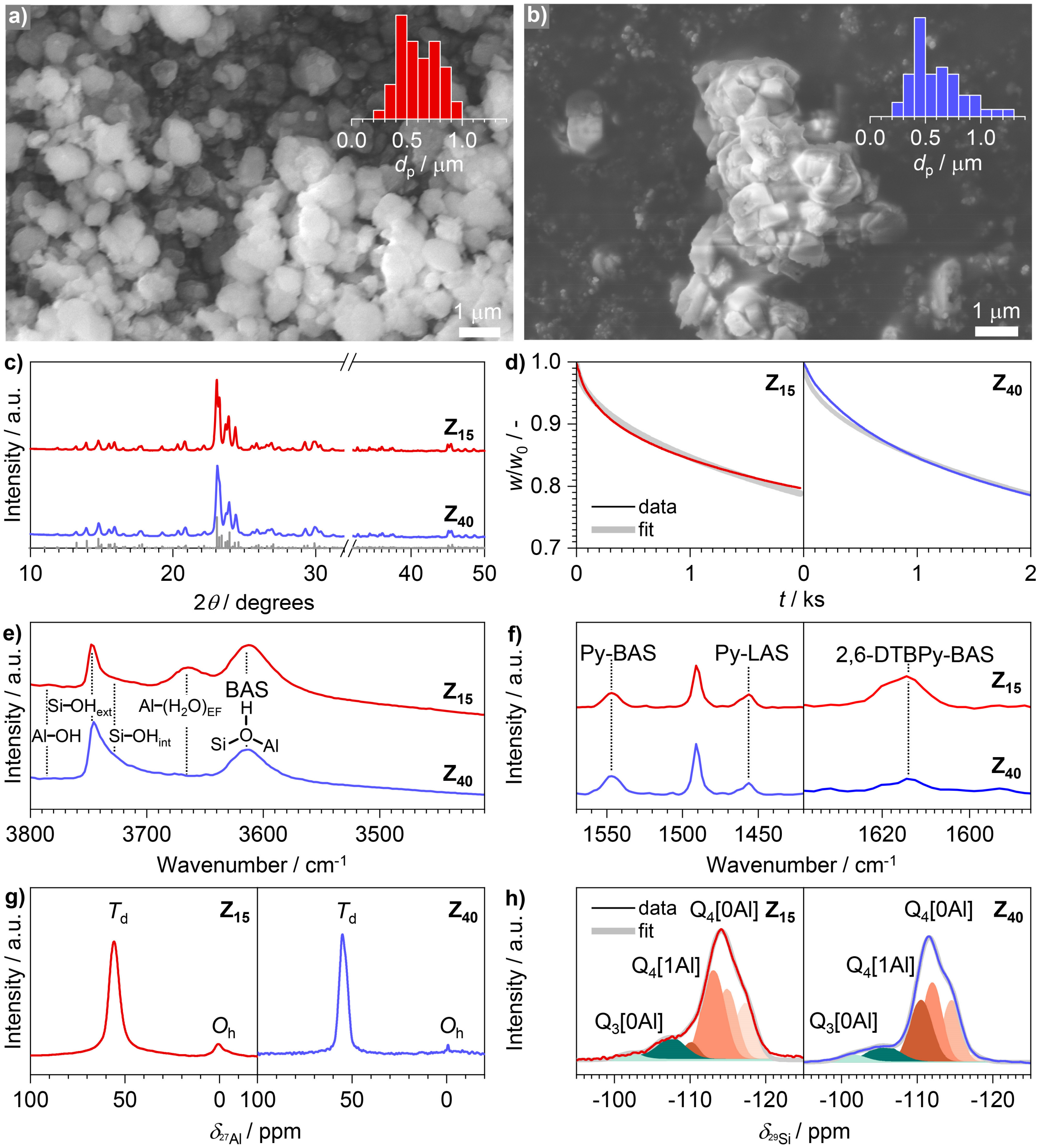

Figure S1. a,b) Scanning electron microscopy (SEM) images and the particle size distribution, c) powder X-ray diffractograms, d) toluene desorption thermogravimetric profiles, e) O−H stretching region, and f) adsorbed pyridine (Py, left) and 2,6-di-tert-butylpyridine (2,6-DTBPy, right) FTIR spectra, g) ^{27}Al, and h) ^{29}Si magic angle spinning NMR spectra of fresh Z_{15} and Z_{40} catalysts. SEM analysis indicates similar particle size distribution in Z15 and Z40 in the range of ≈0.2-1 μm, while X-ray diffractograms point to high crystallinity of the samples which match the reference MFI reflections (in gray). Toluene desprtion experiments indicate very similar characteristic diffusion time constants ($D_{eff}R^{-2}$) for two catalysts (**Table S1**). The FTIR spectra of the O-H region indicate that intensity of the signal associated with BAS (*ca*. 3610 cm^{-1}) is higher in Z_{15} than in Z_{40}. The spectra of Z_{15} display increased

intensity of the bands associated with extra-framework or framework associated aluminum species: Al-(H_2O)$_{EF}$ (3667 cm^{-1}) as well as Al-OH (3790 cm^{-1}) with respect to Z_{40}.[23] The FTIR spectra acquired after pyridine adsorption exhibit the characteristic bands of the internal modes of pyridine interacting with Lewis acid sites (LAS, 1456 cm^{-1}) and BAS (1490 cm^{-1}, 1546 cm^{-1}).[24] The integrals of the 1546 cm^{-1} and 1456 cm^{-1} indicate a higher concentration of BAS and LAS in Z_{15} than in Z_{40} (**Table S1**). The FTIR spectra collected after adsorption of 2,6-di-tert-butylpyridine, which is a probe sensitive to the surface acid site concentration in ZSM-5, show a characteristic band at 1615 cm^{-1} corresponding to its interaction with BAS. The integral of this band indicates a higher BAS concentration in Z_{15} than in Z_{40} (**Table S1**). The ^{27}Al MAS NMR spectra display an intense resonance at *ca.* 56 ppm, which is ascribed to the tetrahedral (T_d), i.e., framework aluminum sites, and resonance at *ca.* 0 ppm of lower intensity, which corresponds to octahedral (O_h) aluminum sites. The latter spectral feature is more pronounced in Z_{15}, indicating a high concentration of extra-framework aluminum site sites in this sample. ^{29}Si MAS NMR spectra exhibit a high intensity of the signal arising from silicon atoms surrounded only with other silicon atoms in their first shell (Q_4[0Al], deconvoluted into four components in the range of ≈-111 to -120 ppm), a low intensity shoulder associated with silicon atoms with one nearest aluminum neighbor (Q_4[1Al], ≈-106.5 ppm), followed by a weak signal assigned to defect Si sites (Q_3[0Al], ≈-102 ppm).[25–27] Deconvolution of ^{29}Si MAS NMR spectra indicates that fresh Z_{15} and Z_{40} catalyst exhibit the Si/Al ratios of 22 and 39 (**Table S1**), respectively, which is consistent with previous reports on these materials.[27]

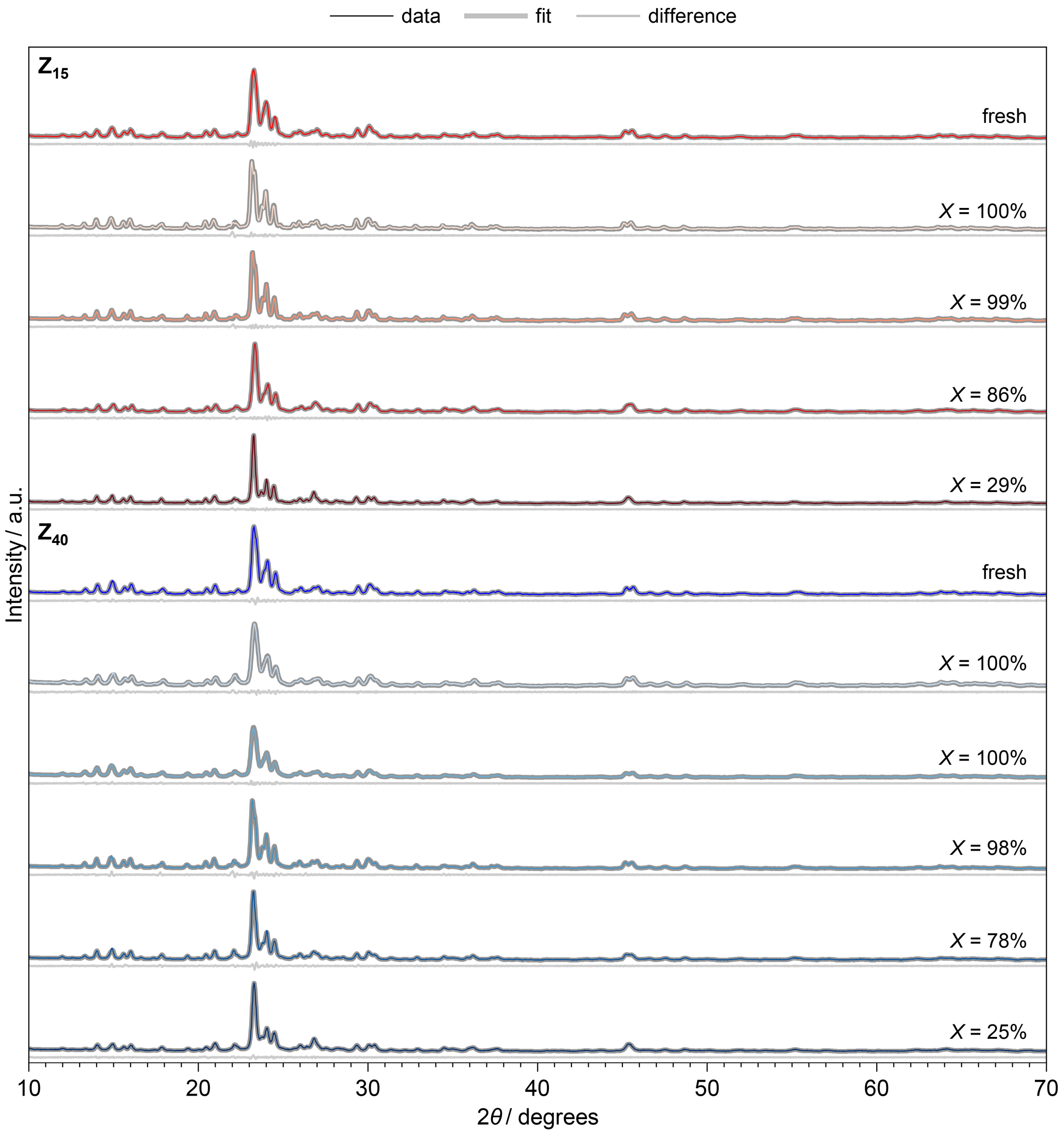

Figure S2. Pawley refinement of XRD data of Z_{15} and Z_{40} in their fresh and deactivated forms. The lattice parameters were determined by refining the orthorhombic *Pnma* space group model of the ZSM-5 zeolite (MFI topology). Deactivated Z_{15} and Z_{40} were retrieved at different stages of deactivation as indicated by residual conversion (X) from the experiments performed under conditions equivalent to those reported in **Figure 1a,b** of the main manuscript. The deactivation of ZSM-5 catalysts results in changes in their diffraction peak positions, indicating a contraction of $\boldsymbol{a}$ and an expansion of $\boldsymbol{b}$ lattice parameters, respectively (**Table S2**). These changes are primarily associated with the accumulation of internal coke that induces lattice macrostrain. For similar coke content, the deformation of orthorhombic lattice, measured as the relative evolution of a difference between $\boldsymbol{a}$ and $\boldsymbol{b}$ unit cell parameters with

respect to the fresh samples,[28] is more pronounced for Z_{15} catalyst (**Table S2**). Assuming the deformation primarily originates from molecules within the micropores, this observation supports a greater accumulation of internal coke in more acidic Z_{15} compared to less acidic counterpart.

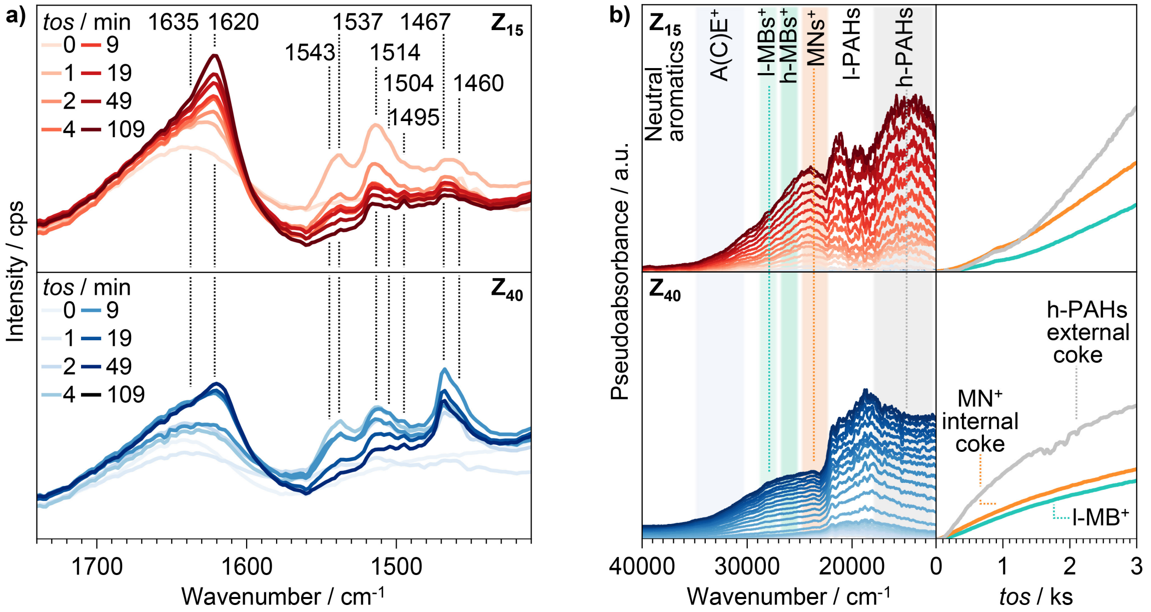

Figure S3. a) *In situ* FTIR and b) *operando* DR-UV-vis spectra during MTH reaction over Z_{15} and Z_{40} catalysts. Reaction conditions: a) CH_3OH = 100 mol%, T = 773 K, and P = 1.2 × 10^{-3} bar. b) CH_3OH:Ar = 0.5:99.5 mol%, WHSV = 4.5 g_{CH3OH} g_{cat}^{-1} h^{-1}, T = 773 K, and P = 1.2 bar. Deactivation of two ZSM-5 zeolites was additionally analyzed by the *in situ* FTIR analysis and *operando* DR-UV-vis experiments, which were performed in the millibar range. FTIR spectra display the bands at ≈ 1635 and ≈ 1620 cm^{-1}, which are attributed to C=C bond vibrations in alkene and methylated aromatic species (**Figure S3a**).[29,30] Additionally, the bands centered at 1543, 1537, 1514, 1509, and 1495 cm^{-1}, ascribed to C=C-C^+ vibrations of alkyl cyclopentenyl cations, as well as the bands at 1467 and 1460 cm^{-1}, attributed to methyl deformations in these species are also detectable. The intensity of the bands arising from the alkyl cyclopentenyl cations, which are considered as highly active HP carriers and intermediates between alkene and arene cycle, decreases more prominently in the spectrum of Z_{15} than in the one of Z_{40}. This coincides with faster increase of the arene-associated bands in the spectrum of Z_{15}, corroborating its faster deactivation. In analogy to Raman spectra (**Figure 1e** of the main manuscript), the peak centered at 1620 cm^{-1} has a more prominent tail towards the lower wavenumbers, suggesting the higher presence of more condensed, graphite-like coke species. The *operando* DR-UV-vis spectra collected at the onset of the MTH reaction over two ZSM-5 catalysts exhibit prominent bands at ≈ 27500 cm^{-1} and ≈ 26000 cm^{-1}, ascribed to methylbenzenium cations with low (l-MB^+) and high (h-MB^+) methylation extent that are the central chain carriers in the aromatic part of cycle (**Figure S3b**).[31,32] The contributions of the bands at ≈ 32000 cm^{-1}, attributed to alkylated (cyclo)enyl cations are more

prevalent in Z_{40} than in Z_{15} catalyst. The bands at ≈ 23500, ≈ 22000-18000, and < 18000 cm^{-1}, attributed to methylnapthalenium ions (MN^+), and PAHs of lower (l-PAHs) and higher (h-PAHs) molecular weight promptly accompany the formation of HP active species over both catalysts. While the-h-PAHs- and MN^+-associated spectral components increase at similar intensity over Z_{15}, the former component increases much more prominently than the latter over Z_{40}. Since MN^+ species can be considered as representatives of internal coke, while the h-PAHs are preferentially depositing on the outside catalyst surface, these results indicate a higher preference of a less acidic catalyst to external coking,[32] which is consistent with the experiments performed at increased methanol pressures.

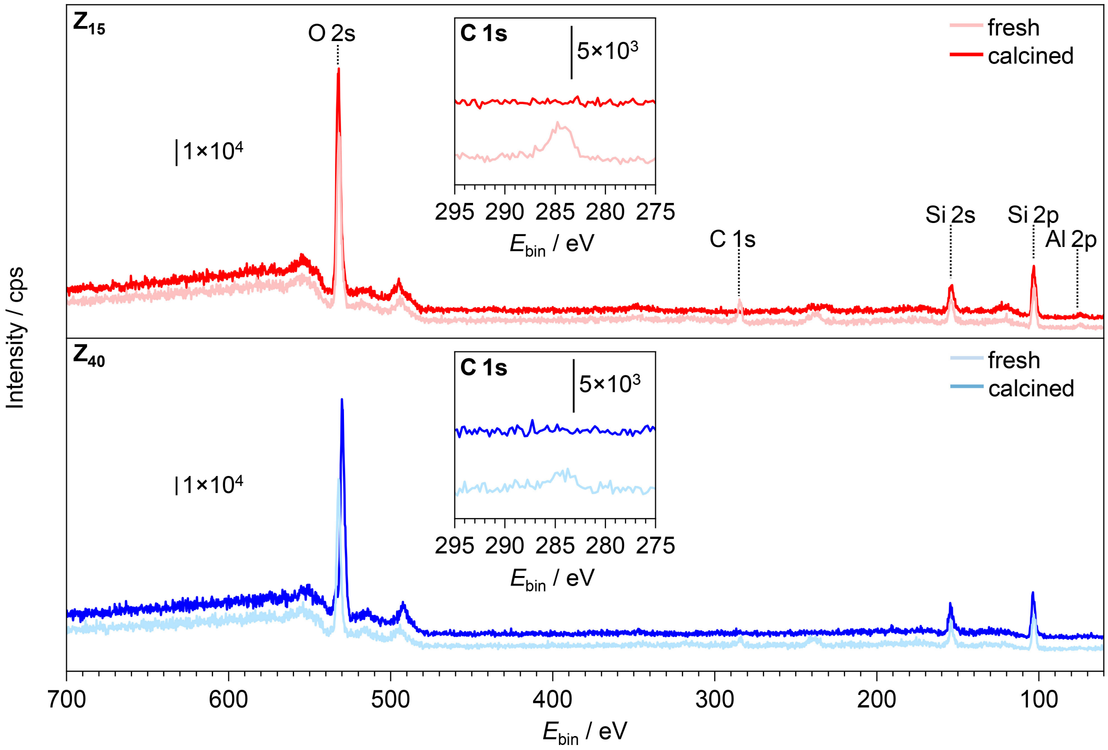

Figure S4. Survey X-ray photoemission spectra of Z_{15} and Z_{40} catalysts recorded at excitation energy of *hv* = 1000 eV. The survey spectra, referenced to the Si 2p binding energy (E_{bin}) of 103.5 eV, show Al 1s, Si 2p, O 2s, and relatively weak Al 2p peaks, as expected for ZSM-5 zeolites. While the spectra of just loaded fresh zeolites exhibit a minor C 1s signal arising from adventitious carbon, the spectra after calcination under oxygen flow show no detectable signals of carbon-containing species (insets). This confirms that the C 1s signal recorded upon methanol dosing and reaction at 473 K and fast heating to 773 K, originates exclusively from reaction-associated species.

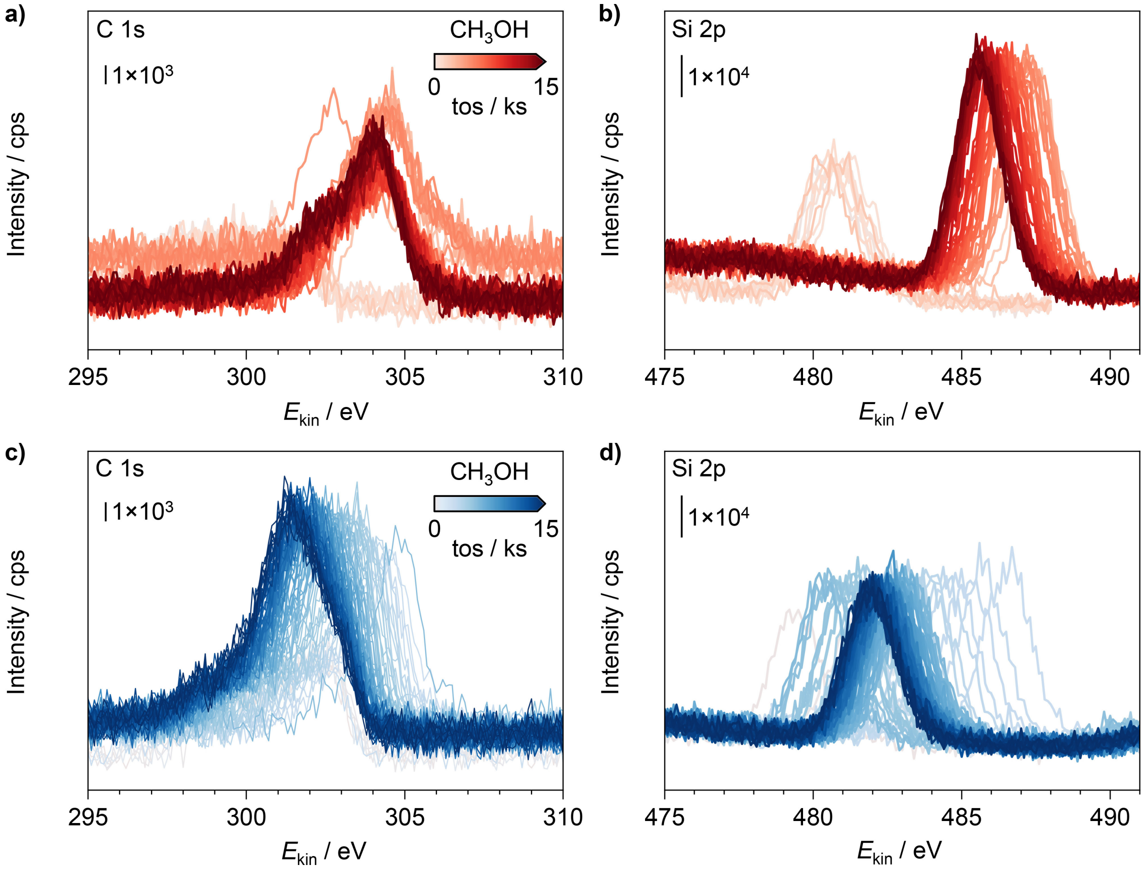

Figure S5. *In situ* C 1s and Si 2p X-ray photoemission spectra recorded during methanol reaction over a,b) Z_{15} and c,d) Z_{40} catalysts at electron kinetic energy of carbon of E_{kin} = 300 eV. Reaction conditions correspond to those used in **Figure 2** of the main manuscript. The X-ray photoemission spectra acquisition is inevitably accompanied by sample charging, arising from the semiconducting nature of zeolites, which is a common property of virtually all realistic catalytic materials. It is important to distinguish between surface charging, caused by charge accumulation at the surface upon photoemission, and differential charging, where different areas of a sample surface accumulate different amounts of electrical charge. While the first leads to a uniform shift (negative shift in kinetic energy and positive in binding energy), the second leads to non-uniform peak shifts and significant peak broadening due to multiple components within a small kinetic energy range. Although effects were reduced by dispersing the zeolite over a conductive silver mesh and maintaining gas flow over the sample, the C 1s spectra still displays a non-monotonic shift of E_{kin} with temperature (**Figure S5a,c**), necessitating an appropriate alignment of the energy scale. To address this, Si 2p spectra were acquired in parallel with C 1s spectra (**Figure S5,d**). The Si 2p signal was then used as an internal reference of C 1s spectra by setting its binding energy to E_{bin} = 103.5 eV, which corresponds to the average of typically reported values for zeolites and silica-like materials.[10] While this approach introduces some uncertainty upon assigning different C 1s spectra components, these uncertainties are expected to remain within ≈ 0.5 eV and to affect both Z_{15} and Z_{40} equally. Considering also the variability in literature reported reference values for carbon containing compounds (**Table S3**), this has only a minor influence on the analysis of spectral changes during the MTH reaction. Because coke is homogeneously dispersed over the samples and thus in contact with the crystals, it is reasonable to assume that Si and C share the same surface

charging. Importantly, the Si 2p spectra acquired in parallel with the respective C 1s spectra consistently display a single doublet characteristic of silicon in zeolites, without any broadening that would indicate differentialcharging effects. Additionally, as shown by survey spectra (**Figure S4**) we did not detect any signal from the metal grid support, which could cause different C 1s charging because of its presence on both the metallic mesh (conductive) and on the zeolite (semiconductive). Moreover, after referencing the E_{bin} scale, the main features in C 1s spectra are well aligned upon data processing (**Figure 2** of the main manuscript), showing features that evidence the formation of unsaturated, graphitic coke formation. These observations rule out the influence of differential charging effects in the C 1s spectra.

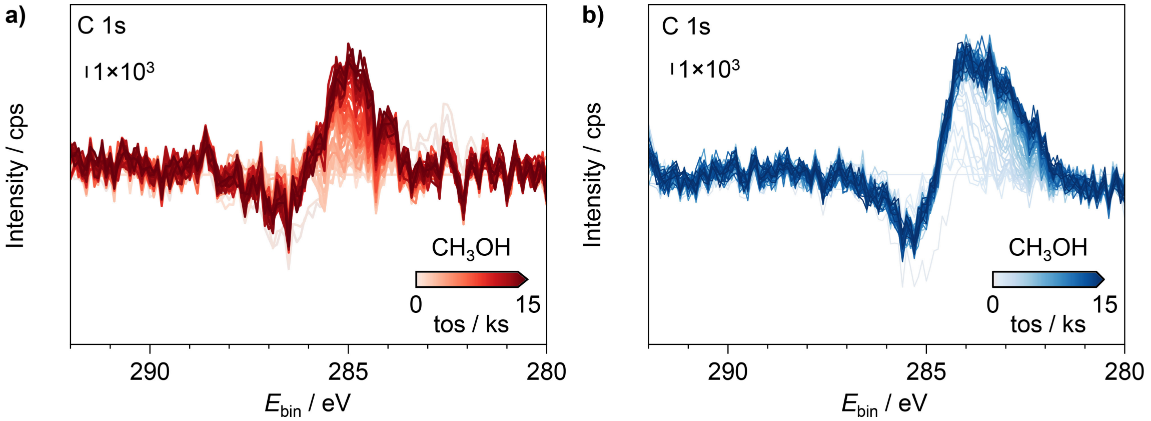

Figure S6. Difference *in situ* C 1s X-ray photoemission spectra during methanol reaction over a) Z_{15} and b) Z_{40} catalysts upon heating up from 473 K to 773 K at an electron kinetic energy of carbon of E_{kin} = 300 eV. Reaction conditions correspond to those used in **Figure 2** of the main manuscript. To visually highlight the spectral regions exhibiting the most pronounced changes during methanol conversion, the difference spectra were calculated by subtracting the measured, Si 2*p* binding-energy-corrected spectra (**Figure S5**) from the corresponding reference spectra acquired in the initial stage of the experiment. For Z_{15}, the difference C 1*s* spectra reveal an initially rapid decrease in intensity in the E_{bin} ≈ 286-287 eV, accompanied by a progressive increase in intensity at E_{bin} ≈ 284-285.5 eV. In Z_{40}, a fast and negative intensity change is observed at E_{bin} ≈ 285-286.5 eV in Z_{40}, which is coupled with the steady positive intensity increase in the range of E_{bin} ≈ 282-284.5 eV. This increase becomes more pronounced at the lower end of the binding-energy region as the reaction proceeds. Based on spectral assignments (**Table S3**), the difference spectra indicate the conversion of oxygenates into hydrocarbon species, followed by possible interconversion of the hydrocarbon species, which is most prominent in Z_{40}.

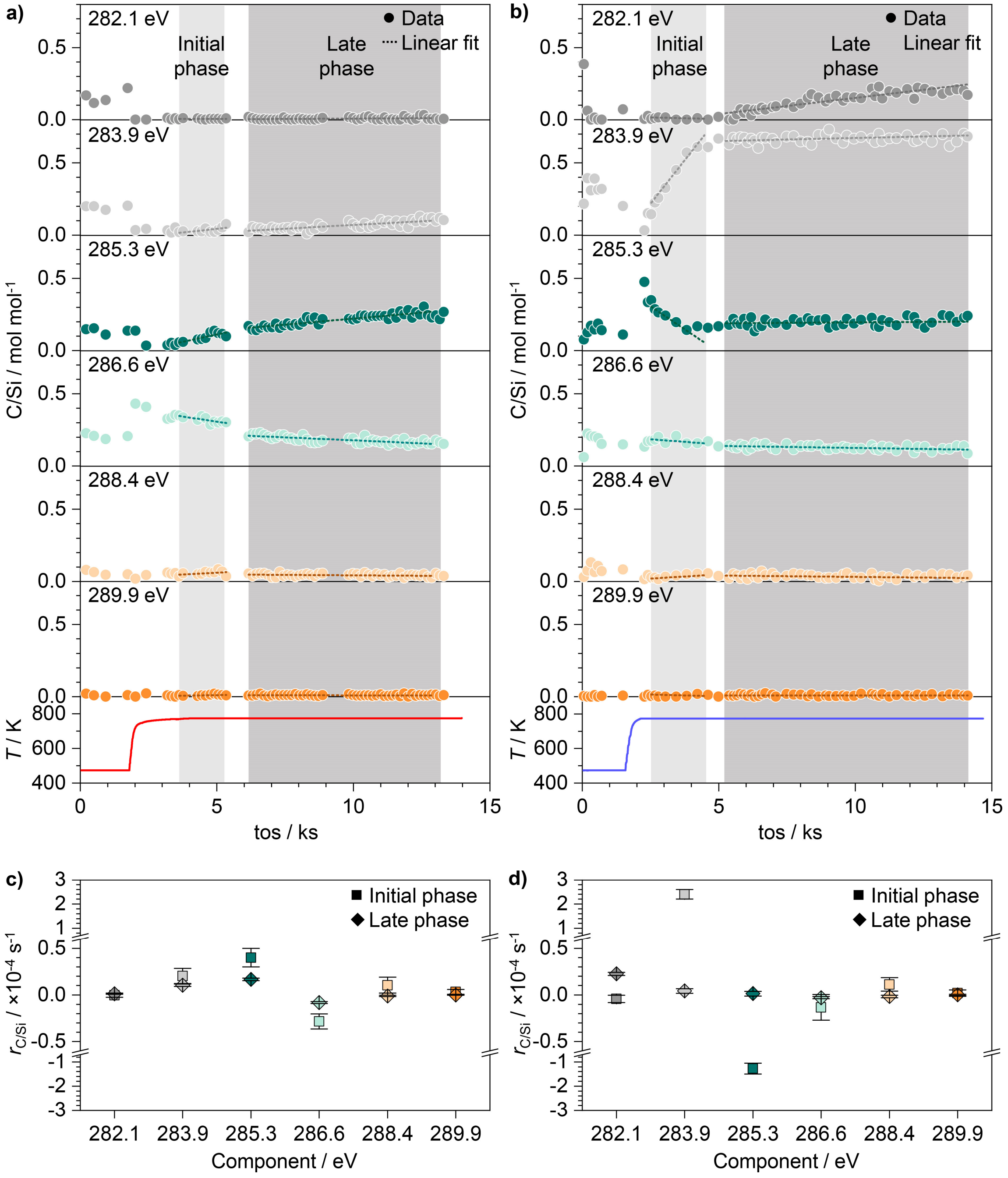

Figure S7. a,b) The time profiles of spectral components in the *in situ* C 1s X-ray photoemission spectra and c,d) extracted rates ($r_{C/Si}$) during methanol reaction over a,c) Z_{15} and b,d) Z_{40} catalyst. The error bars in c,d) present the 95% confidence interval of the fitted slopes. Reaction conditions correspond to those used in Figure 2 of the main manuscript. The temporal evolution of the spectral components reveals an initial phase characterized by rapid intensity changes, followed by a late phase with markedly slower evolution. Linear fitting of the data in these two regimes (**Figure S6a,b**) provides estimates of the rates of change of the individual components (**Figure S6a,b**). In Z_{15}, the most intense component at E_{bin} ≈ 286.6 eV, attributed to oxygen-containing species decreases at the highest rate (≈3×10^{-5} s^{-1}) in the initial phase. This is accompanied with the growth of hydrocarbon-related components at E_{bin} ≈ 285.3 (≈4×10^{-5} s^{-1}) and 283.9 eV (≈2×10^{-5} s^{-1}), with the former increasing more rapidly in both

reaction phases. In the initial phase over Z_{40}, the hydrocarbon component at $E_{bin} \approx 285.3$ eV decays at the highest rate (≈1.3×10^{-4} s^{-1}), while another hydrocarbon component at $E_{bin} \approx 283.9$ eV exhibits the highest rate of growth (≈2.4×10^{-4} s^{-1}). However, in the late phase, both change at very low rate (in the range of ≈1×10^{-6} s^{-1}). The central characteristic of Z_{40} compared to Z_{15} is the appearance of the component at $E_{bin} \approx 282.1$ eV, which can be associated with highly dehydrogenated, graphite-like coke species.[11,15] Notably, this component increases most prominently (≈2.3×10^{-5} s^{-1}) in the later reaction phase, dominating the carbon buildup. These kinetic profiles indicate distinct coke-formation pathways, with coke in Z_{15} arising primarily from oxygenate conversion, whereas in Z_{40} it predominantly forms through the secondary transformation of hydrocarbon species into graphitic coke.

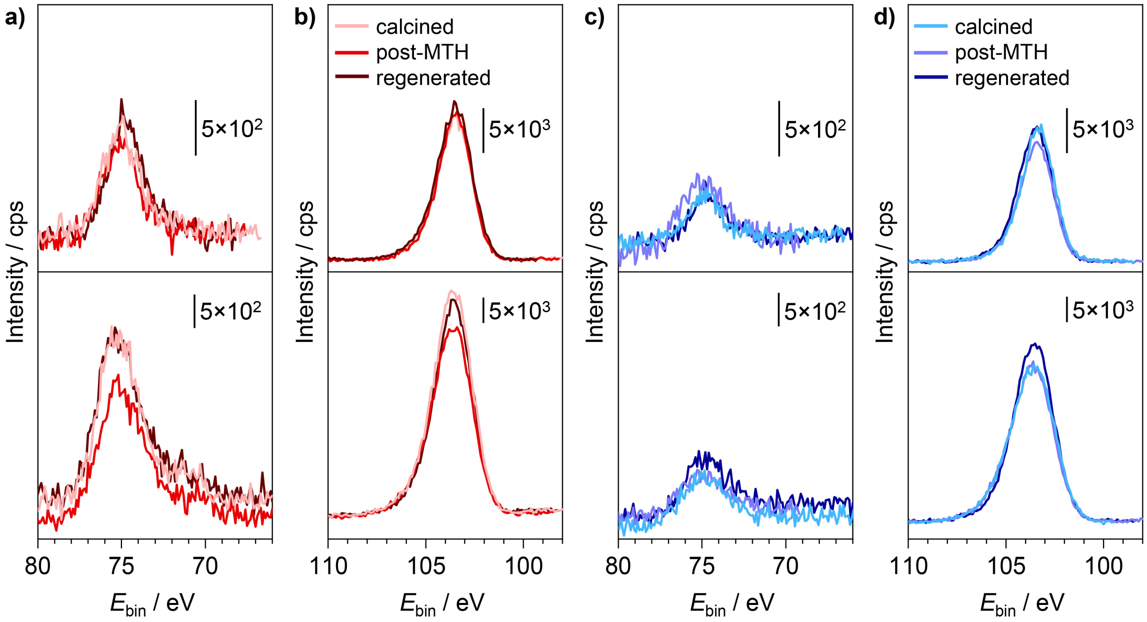

Figure S8. *In situ* Al 2p and Si 2p X-ray photoemission spectra of fresh, deactivated and regenerated a,b) Z_{15} and c,d) Z_{40} catalysts at electron kinetic energies of E_{kin} = 300 (top) and 600 eV (bottom), used for estimating Si/Al ratios. The spectra were collected after calcination, after methanol reaction, and regeneration under Ar flow at 473 K. Other reaction conditions correspond to those used in **Figure 2** of the main manuscript. The integrals of collected spectra were used to calculate the surface Si/Al ratios in fresh, post-MTH and regenerated catalysts according to **Equations S7,S8** (**Tables S1,S4**).

SI References

[1] A. A. Coelho, "TOPAS and TOPAS-Academic: An optimization program integrating computer algebra and crystallographic objects written in C++: An" *J. Appl. Crystallogr.* **2018**, *51*, 210–218.

[2] L. Milaković, P. H. Hintermeier, Y. Liu, E. Baráth, J. A. Lercher, "Influence of Intracrystalline Ionic Strength in MFI Zeolites on Aqueous Phase Dehydration of Methylcyclohexanols" *Angew. Chem. Int. Ed.* **2021**, *60*, 24806–24810.

[3] K. Góra-Marek, K. Tarach, M. Choi, "2,6-Di-tert-butylpyridine sorption approach to quantify the external acidity in hierarchical zeolites" *J. Phys. Chem. C* **2014**, *118*, 12266–12274.

[4] J. Crank, *The Mathematics of Diffusion*, Oxford University Press, Oxford, **1975**.

[5] V. Paunović, P. Hemberger, A. Bodi, R. Hauert, J. A. van Bokhoven, "Impact of Nonzeolite-Catalyzed Formation of Formaldehyde on the Methanol-to-Hydrocarbons Conversion" *ACS Catal.* **2022**, *12*, 13426–13434.

[6] F. Orlando, A. Waldner, T. Bartels-Rausch, M. Birrer, S. Kato, M. T. Lee, C. Proff, T. Huthwelker, A. Kleibert, J. Van Bokhoven, M. Ammann, "The Environmental Photochemistry of Oxide Surfaces and the Nature of Frozen Salt Solutions: A New in Situ XPS Approach" *Top. Catal.* **2016**, *59*, 591–604.

[7] L. Artiglia, F. Orlando, K. Roy, R. Kopelent, O. Safonova, M. Nachtegaal, T. Huthwelker, J. A. Van Bokhoven, "Introducing Time Resolution to Detect Ce^{3+} Catalytically Active Sites at the Pt/CeO2 Interface through Ambient Pressure X-ray Photoelectron Spectroscopy" *J. Phys. Chem. Lett.* **2017**, *8*, 102–108.

[8] M. Guo, N. Dongfang, M. Iannuzzi, J. A. van Bokhoven, L. Artiglia, "Structure and Reactivity of Active Oxygen Species on Silver Surfaces for Ethylene Epoxidation" *ACS Catal.* **2024**, *14*, 10234–10244.

[9] C. J. Powell, A. Jablonski, "NIST Electron Inelastic-Mean-Free-Path Database" **2000**, 2000.

[10] J. F. Moulder, W. F. Stickle, P. E. Sobol, K. D. Bomben, *Handbook of X-ray Photoelectron Spectroscopy*, Perkin-Elmer Corp., Eden Prairie, **1992**.

[11] A. . Bhattacharya, D. . Pyke, G. . Walker, C. . Werrett, "The Surface Reactivity of Different Aluminas as Revealed by Their XPS C 1*s* Spectra" *Appl. Surf. Sci.* **1997**, *108*, 465–470.

[12] R. Davì, G. Carraro, M. Stojkovska, M. Smerieri, L. Savio, M. Lewandowski, J. J. Gallet, F. Bournel, M. Rocca, L. Vattuone, "Graphene growth on Ni (1 1 1) by CO Exposure at Near Ambient Pressure" *Chem. Phys. Lett.* **2021**, *774*, 138596.

[13] S. A. Steiner, T. F. Baumann, B. C. Bayer, R. Blume, M. A. Worsley, W. J. MoberlyChan, E. L. Shaw, R. Schlögl, A. J. Hart, S. Hofmann, B. L. Wardle, "Nanoscale Zirconia as a Nonmetallic Catalyst for Graphitization of Carbon and Growth of Single- and Multiwall Carbon Nanotubes" *J. Am. Chem. Soc.* **2009**, *131*, 12144–12154.

[14] L. Hohmann, F. Dahlmann, G. B. Braghin, L. Laviron, L. Hussein, J. Martinez, A. Harrer, H. Robertson, J. Guiborat, X. Hu, J. Weissenrieder, K. Engvall, J. LaRue, T. Hansson, M. Göthelid, A. Ghassami, D. J. Harding, H. Öström, “Naphthalene Decomposition on Fe(110)─Adsorption, Dehydrogenation, Surface Carbon Formation and the Influence of Coadsorbed Oxygen” *J. Phys. Chem. C* **2025**, *129*, 2441–2452.

[15] B. M. Weckhuysen, M. P. Rosynek, J. H. Lunsford, “Characterization of Surface Carbon Formed during the Conversion of Methane to Benzene over Mo/H-ZSM-5 Catalysts” *Catal. Letters* **1998**, *52*, 31–36.

[16] J. C. Scott, “Metal–Organic Interface and Charge Injection in Organic Electronic Devices” *J. Vac. Sci. Technol. A Vacuum, Surfaces, Film.* **2003**, *21*, 521–531.

[17] A. F. Lee, K. Wilson, A. Goldoni, R. Larciprete, S. Lizzit, “A Fast XPS Study of Propene Decomposition over Clean and Sulphated Pt{111}” *Catal. Letters* **2002**, *78*, 379–382.

[18] M. Morkel, V. V. Kaichev, G. Rupprechter, H. J. Freund, I. P. Prosvirin, V. I. Bukhtiyarov, “Methanol Dehydrogenation and Formation of Carbonaceous Overlayers on Pd(111) Studied by High-Pressure SFG and XPS Spectroscopy” *J. Phys. Chem. B* **2004**, *108*, 12955–12961.

[19] A. F. Lee, K. Wilson, R. L. Middleton, A. Baraldi, A. Goldoni, G. Paolucci, R. M. Lambert, “In Situ Observation of a Surface Chemical Reaction by Fast X-Ray Photoelectron Spectroscopy” *J. Am. Chem. Soc.* **1999**, *121*, 7969–7970.

[20] Y. Katayama, R. Kubota, R. R. Rao, J. Hwang, L. Giordano, A. Morinaga, T. Okanishi, H. Muroyama, T. Matsui, Y. Shao-Horn, K. Eguchi, “Direct Observation of Surface-Bound Intermediates During Methanol Oxidation on Platinum Under Alkaline Conditions” *J. Phys. Chem. C* **2021**, *125*, 26321–26331.

[21] S. Reiche, R. Blume, X. C. Zhao, D. Su, E. Kunkes, M. Behrens, R. Schlögl, “Reactivity of Mesoporous Carbon against Water - An In Situ XPS Study” *Carbon N. Y.* **2014**, *77*, 175–183.

[22] A. F. Lee, D. E. Gawthrope, N. J. Hart, K. Wilson, “A Fast XPS Study of the Surface Chemistry of Ethanol over Pt{111}” *Surf. Sci.* **2004**, *548*, 200–208.

[23] L. Treps, C. Demaret, D. Wisser, B. Harbuzaru, A. Méthivier, E. Guillon, D. V. Benedis, A. Gomez, T. De Bruin, M. Rivallan, L. Catita, A. Lesage, C. Chizallet, “Spectroscopic Expression of the External Surface Sites of H-ZSM-5” *J. Phys. Chem. C* **2021**, *125*, 2163–2181.

[24] S. Bordiga, C. Lamberti, F. Bonino, A. Travert, F. Thibault-Starzyk, “Probing Zeolites by Vibrational Spectroscopies” *Chem. Soc. Rev.* **2015**, *44*, 7262–7341.

[25] T. Liang, J. Chen, Z. Qin, J. Li, P. Wang, S. Wang, G. Wang, M. Dong, W. Fan, J. Wang, “Conversion of Methanol to Olefins over H-ZSM-5 Zeolite: Reaction Pathway Is Related to the Framework Aluminum Siting” *ACS Catal.* **2016**, *6*, 7311–7325.

[26] L.-H. He, J.-J. Li, S.-Y. Han, D. Fan, X.-J. Li, S.-T. Xu, Y.-X. Wei, Z.-M. Liu, “Dynamic Evolution

of HZSM-5 Zeolite Framework under Steam Treatment" *Chem. Synth.* **2023**, *3*, 1–18.

[27] J. Holzinger, P. Beato, L. F. Lundegaard, J. Skibsted, "Distribution of Aluminum over the Tetrahedral Sites in ZSM-5 Zeolites and Their Evolution after Steam Treatment" *J. Phys. Chem. C* **2018**, *122*, 15595–15613.

[28] D. Rojo-Gama, M. Nielsen, D. S. Wragg, M. Dyballa, J. Holzinger, H. Falsig, L. F. Lundegaard, P. Beato, R. Y. Brogaard, K. P. Lillerud, U. Olsbye, S. Svelle, "A Straightforward Descriptor for the Deactivation of Zeolite Catalyst H-ZSM-5" *ACS Catal.* **2017**, *7*, 8235–8246.

[29] I. B. Minova, S. K. Matam, A. Greenaway, C. R. A. Catlow, M. D. Frogley, G. Cinque, P. A. Wright, R. F. Howe, "Elementary Steps in the Formation of Hydrocarbons from Surface Methoxy Groups in HZSM-5 Seen by Synchrotron Infrared Microspectroscopy" *ACS Catal.* **2019**, *9*, 6564–6570.

[30] S. M. Auerbach, F. C. Jentoft, E. D. Hernandez, B. Manookian, "Shape-Selective Synthesis of Alkylcyclopentenyl Cations in Zeolites and Spectroscopic Distinction of Constitutional Isomers" *ACS Catal.* **2021**, *11*, 12893–12914.

[31] J. Goetze, I. Yarulina, J. Gascon, F. Kapteijn, B. M. Weckhuysen, "Revealing Lattice Expansion of Small-Pore Zeolite Catalysts during the Methanol-to-Olefins Process Using Combined Operando X-ray Diffraction and UV–vis Spectroscopy" *ACS Catal.* **2018**, *8*, 2060–2070.

[32] D. Fu, O. Heijden, K. Stanciakova, J. E. Schmidt, B. M. Weckhuysen, "Disentangling Reaction Processes of Zeolites within Single-Oriented Channels" *Angew. Chem. Int. Ed.* **2020**, *59*, 15502–15506.
